# Free and bound phenolic profiles and antioxidant ability of eleven marine macroalgae from the South China Sea

**DOI:** 10.3389/fnut.2024.1459757

**Published:** 2024-10-14

**Authors:** Ziting Peng, Yujiao Wu, Qiongyao Fu, Juan Xiao

**Affiliations:** ^1^National Health Commission of the People’s Republic of China, Key Laboratory of Control of Tropical Diseases Control, School of Tropical Medicine, Hainan Medical University, Haikou, Hainan, China; ^2^Hainan Engineering Research Center of Aquatic Resources Efficient Utilization in South China Sea, Key Laboratory of Seafood Processing of Haikou, Key Laboratory of Food Nutrition and Functional Food of Hainan Province, School of Food Science and Engineering, Hainan University, Haikou, China

**Keywords:** marine algae, bound phenolic, phenolic profile, antioxidant ability, total phenolic content, total phlorotannin content, total flavonoid content

## Abstract

Marine macroalgae are of broad interest because of their abundant bioactive phenolic compounds. However, only a few previous studies have focused on bound phenolic compounds. In this study, there were significant differences in total phenolic content, total phlorotannin content, total flavonoid content, and antioxidant ability in free and bound forms, as well as in their bound-to-free ratios, among 11 marine macroalgal species from the South China Sea. *Padina gymnospora* had the highest total phenolic content of free fractions, and total phlorotannin content, total flavonoid content, and antioxidant activity of free fractions. *Sargassum thunbergii* had the highest total phlorotannin content of bound fractions, whereas *Sargassum oligocystum* had the highest total flavonoid content and total phenolic content of bound fractions. Moreover, 15 phenolic acids, 35 flavonoids, 2 stilbenes, 3 bromophenols, and 3 phlorotannins were characterized and quantified using ultra-high-performance liquid chromatography with Xevo triple quadrupole mass spectrometry, and 42 phenolic compounds were reported in the bound fractions of seaweeds for the first time. Among the species, the number and amount of free and bound phenolic compounds varied greatly and the main components were different. *Padina gymnospora* had the largest total phenolic number, while *Turbinaria ornata* showed the highest total phenolic amount. Coutaric acid and diosmetin were dominant in *Sargassum polycystum*, and hinokiflavone was dominant in *Caulerpa lentillifera*, and cyanidin was dominant in the other seaweeds. Hierarchical cluster analysis was used to divide the seaweed species into seven groups. This study revealed that *Padina gymnospora*, *Sargassum thunbergii*, *Turbinaria ornata*, and *Sargassum oligocystum* are promising functional food resources.

## 1 Introduction

Marine macroalgae, also known as seaweeds, are an alternative food source because of their abundance of nutrients, such as proteins, fatty acids, and dietary fiber. Seaweed is an important source of bioactive metabolites (1). Depending on the pigments and characteristic metabolites, seaweeds are usually divided into Phaeophyceae (brown seaweeds), Chlorophyta (green seaweeds), and Rhodophyta (red seaweeds) (2). Accumulating studies have demonstrated that brown, green and red seaweeds exert potent biological activities including antioxidant, anti-inflammatory, antibacterial, anti-hyperglycemia and hepatoprotective activities (2, 3), which are closely associated with the bioactive metabolites contained in seaweeds. Therefore, seaweeds have emerged as an important resource because of their potential commercial value in the food, pharmaceutical, and other fields.

Phenolic compounds are ubiquitous throughout the plant kingdom including terrestrial plants and seaweeds (2). Among marine bioactive metabolites, phenolic compounds compose the largest family of secondary metabolites (2, 4). The biosynthesis of phenolic compounds in seaweeds is related to environmental stressors including salinity, desiccation, and ultraviolet radiation, which contributes to the great diversity in the phenolic profiles of seaweeds (5). The significant difference in environmental stressors between terrestrial plants and seaweeds is partly responsible for the discrepancy in their phenolic profiles (5). The structure of phenolic compounds is comprised of an aromatic ring with at least one hydroxyl group and/or other groups (6). The simplest phenolic compounds, phenolic acids, are not exclusive to terrestrial plants, as they also exist in seaweeds (2). Although flavonoids are found in both terrestrial plants and seaweeds, the glycosylated forms instead of the aglycones are usually characterized in seaweeds (1, 4). Catechins, especially epicatechin and epigallocatechin derivations, are present in seaweeds (7). In comparison to phenolic acids, flavonoids, and catechins, phlorotannins and bromophenols are usually considered to be more specific to seaweeds (8). Phlorotannins, the complex polymers of phloroglucinol, are structurally and functionally analogous to condensed tannins derived from terrestrial plants, and are primarily identified in brown algae (1, 3, 9). Bromophenols, which are the characteristic phenolic compounds of red seaweeds, have also been detected in green and brown seaweeds (10). Their aromatic properties are responsible for the flavor of seaweeds (11). Due to the structural diversity and variability, phenolic compounds from seaweeds are still far less explored than terrestrial plants until now (4, 8).

Usually, phenolic compounds exist in free and bound forms in plants (12). The free form is usually present in vacuoles and does not bind to other molecules, resulting in good solubility in polar aqueous or organic solvent (6). The bound form usually binds to macromolecules (such as arabinoxylans, pectin, cellulose, hemicellulose, lignin or proteins) in the cell wall of terrestrial plants (13). The bound phenolic compounds are predominant in cereal grains, whereas the free form is predominant in fruits and vegetables (14, 15). Several phenolic acids are more abundant in the bound fraction of grains and beans (6, 13). Hydrogen bonding and hydrophobic interactions are considered as driving mechanisms for phenolic compounds to bind to carbohydrates (16). There are significant differences in phenolic profiles bound to insoluble and soluble dietary fiber from both defatted rice bran and seaweeds (17, 18). The binding of phenolic acids with protein mainly depends on the molecular weight, structural flexibility, affinity of phenolic acid for water, and hydroxyl groups (19). Considering the obvious difference in cell wall components between terrestrial plants and seaweeds (20), enormous differences may exist in their bound phenolic profiles.

The potent biological activity makes phenolic compounds an attractive group deserving the increasing attention. Free and bound phenolic compounds in terrestrial plants have been shown to exert various biological activities *in vitro* and *in vivo* (12). However, owing to the interaction with cell wall macromolecules, bound phenolic compounds in terrestrial plants have low absorbability in the small intestine (13), and thus they are not paid enough attention in comparison to free phenolic compounds. Similarly, limited data are available on the bound form of seaweeds (18, 21, 22), although an increasing number of studies have also focused on the profile and activity of free phenolics from seaweeds (5, 8, 23, 24). In fact, bound phenolic compounds can be released in the colon by the enzymes secreted by microbiota and are biotransformed by different metabolic pathways. The metabolites showed more potent physiological activities and higher absorbability than the parent compounds, and contributed to the modulation of intestinal microbiota composition (6). Thus, further research on bound phenolic compounds in seaweeds would be highly valuable.

In China, seaweed resources are abundant, with over 1,200 species accounting for approximately 1/6 of the total species globally (25). Approximately 70% of seaweed species in China are distributed in the South China Sea, whereas a small proportion are cultured artificially or commercially valuable for food and food additives, pharmaceuticals, and fertilizers (26). However, various seaweeds have been used in traditional Chinese medicine and local food for many centuries without knowledge of their bioactive compounds (27). With increasing demand for seaweed-derived products in various fields, the profiles of bioactive compounds in seaweeds distributed in the South China Sea are highly valuable.

Therefore, we measured the total phenolic content (TPC), total phlorotannin content (TPhC), and total flavonoid content (TFC) in the free and bound phenolic fractions from 11 seaweed species. The phenolic composition was identified and quantified using ultra-high-performance liquid chromatography with Xevo triple quadrupole mass spectrometry (UPLC-QQQ-MS). Furthermore, the *in vitro* antioxidant activity was measured, and the correlation between antioxidant activity and TPC, TPhC, TFC, and the phenolic profile was analyzed. Differences among different species were evaluated using Principal Component Analysis (PCA) and Hierarchical Cluster Analysis (HCA). The present study provides a scientific basis for the nutritional value of seaweeds and a hint for their application.

## 2 Materials and methods

### 2.1 Materials and reagents

The seaweeds were collected from three different locations: Station 1: Fengjiawan bay, Wenchang, Hainan Province, China (110°51′ 48′′E, 19°31′ 16′′ N), Station 2: Luhuitou, Sanya, Hainan Province (109°48′ 55′′E, 18°21′ 65′′ N), Station 3: Wuzhizhou island, Sanya, Hainan Province (109°45.494′E, 18°18.555’N). Five kinds of Phaeophyta [*Sargassum polycystum* (*S. polycystum*), *Sargassum oligocystum* (*S. oligocystum*), *Sargassum thunbergii* (*S. thunbergii*), *Padina gymnospora* (*P. gymnospora*), *Turbinaria ornata* (*T. ornata*)], four kinds of Rhodophyta [*Asparagopsis taxiformis* (*A. taxiformis*), *Hydropuntia eucheumatoides* Harvey (*H. eucheumatoides*), *Gracilaria tenuistipitata* (*G. tenuistipitata*), *Gloiopeltis furcata* (*G. furcata*)] and two kinds of Chlorophyta [*Caulerpa lentillifera* (*C. lentillifera*), *Caulerpa racemosa* (*C. racemosa*)] were sampled from each area in triplicate. *S. polycystum*, *S. oligocystum*, and *S. thunbergii* were collected from Station 1 in April 2019. *A. taxiformis* was collected from Station 3 in May 2020. The remaining seaweeds were collected from station 2 in June 2020. Fresh seaweeds were collected, identified by Prof. Xiubao Li from the College of Marine Science, Hainan University, packed into plastic bags, kept in an ice-covered polystyrene thermic box and then moved to the laboratory in 12 h. Fresh seaweeds were rinsed with fresh water, freeze-dried, ground, and then the dry seaweeds powder were stored at −18°C for further extraction. The moisture content of the dry seaweeds powder was measured by oven-drying to constant weight at 105°C described by AOAC (28) and used to calculate the weight of dry basis of seaweed. The chemical reagents used in the UPLC-QQQ-MS were of HPLC grade, and the others were of analytical grade.

### 2.2 Extraction of free and bound phenolics

Free phenolic fractions were extracted as described previously (21). Dry seaweed powder (2 g) was homogenized using a XHF-D homogenizer (IKA-Labortechnik, Staufen, Germany) at 5000 rmp with 70% ethanol (60 mL) for 5 min at 4°C and then centrifuged using a H2050R high-speed freezing centrifuge (Hunan Xiangyi Laboratory Instrument Development Co., Ltd., Hunan, China) at 8000 rpm for 10 min at 4°C. The residue was extracted again with 60 mL of 70% ethanol for 5 min at 4°C and then centrifuged. The supernatants obtained from twice extractions were mixed and then vacuum evaporated to dryness at 45°C on a rotating evaporator (RE212-B, Yamatuo Technology Trading Co., Ltd., Chongqing, China), re-dissolved by 85% methanol aqueous solution and filled to the 7 mL mark, and finally stored at −18°C. The extraction was performed in triplicate.

The residue from the free phenolic extract was used to extract bound phenolic compounds using a previously reported alkaline hydrolysis method (21). Briefly, the residue was mixed with 60 mL of NaOH (2 M), subjected to an N_2_ stream for 5 min, and hydrolyzed for 18 h under continuous shake to at room temperature. The mixtures were centrifuged at 10,000 rpm and 4°C for 10 min and the precipitates were repeatedly hydrolyzed and then centrifuged. The supernatants from twice hydrolysis were combined, adjusted to pH 1–2, and then extracted with an equal volume of ethyl acetate using a separatory funnel to perform a liquid–liquid extraction. The organic fractions obtained from five extractions were mixed, vacuum evaporated to dryness at 45°C on a rotating evaporator, re-dissolved by 85% methanol aqueous solution and filled to the 10 mL mark, and finally stored at −18°C. The hydrolysis was performed in triplicate.

### 2.3 Determination of TPC, TPhC, and TFC

The TPC and TFC of the free and bound phenolic fractions were measured using the colorimetric method of Folin–Ciocalteu and NaNO_2_–AlCl_3_, as described previously (21, 29), with gallic acid and catechin as standards. TPC and TFC were expressed as micrograms of gallic acid equivalent (GAE) per 100 g dry basis of seaweed (mg GAE/100 g) and micrograms of catechin equivalent (CE) per 100 g dry basis of seaweed (mg CE/100 g), respectively. TPhC was measured using the Folin–Ciocalteu colorimetric method with slight modifications (3, 9). Briefly, 0.125 mL of the free or bound phenolic fraction was blended with ultrapure water (0.50 mL) and Folin–Ciocalteu reagent (0.125 mL). After 6 min, 7% aqueous sodium carbonate solution (1.25 mL) and ultrapure water (1 mL) were added to the mixture. After 90 min in the dark, absorbance was measured at 760 nm. TPhC was calculated by comparison with a calibration curve of phloroglucinol and expressed as micrograms of phloroglucinol equivalent per 100 g of dry basis of seaweed (mg PGE/100 g). Total TPC, TPhC, and TFC were the sum of TPC, TPhC, and TFC of free and bound fractions, respectively.

### 2.4 Identification and quantification by UHPLC-QQQ-MS

UHPLC-QQQ-MS (Waters, Milford, MA, United States) was used to characterize and quantify phenolics in the free and bound fractions (21). Briefly, an acquity UHPLC BEH-C18 column (2.1 i.d. × 100 mm, 1.7 μm) was eluted by 0.25% formic acid in water (A) and 0.25% formic acid in methanol (B) at a gradient procedure (0–1 min, 5% B; 8 min, 25% B; 11 min, 60% B; 13–16 min, 100% B; 16.2–18 min, 5% B). The compounds were deduced from the data generated from multiple reaction monitoring and compared with the formula mass and mass spectral data from the literature. Subsequently, phenolics were assigned by comparing the retention time, formula mass, and mass spectral data of the phenolic standards with similar basic structures to the aforementioned compounds. Coutaric acid, diphlorethol/difucol, and eckol were quantified using UHPLC-QQQ-MS with calibration curves for gallic acid, phloroglucinol, and phloroglucinol, respectively. Other compounds were quantified using UHPLC-QQQ-MS and their respective standard curves. The content was shown as μg per 10 g of dry basis of seaweed (μg/10 g). Mass spectra data were collected in a mass range of m/z 50–1,000 at 2.0 kV of capillary voltage, 30 V of cone voltage, 1,000 L/h of drying gas (N_2_) flow, and 500°C of drying gas temperature.

### 2.5 Antioxidant activity

The commercial kits with 2,2′-azino-bis (3-ethylbenzthiazoline)-6-sulfonic acid (ABTS) and ferric-reducing antioxidant power (FRAP) assays, purchased from Nanjing Jiancheng Bioengineering Institute (Nanjing, Jiangsu, China), were utilized to measure the antioxidant activity of free and bound fractions according to the manufacturer’s protocols. For ABTS assay, the fractions were mixed with ABTS working solution, reacted at room temperature for 6 min, and then measured at 734 nm. For FRAP assay, the fractions were mixed with fresh FRAP working solution, reacted in dark at room temperature for 30 min, and then measured at 593 nm. ABTS and FRAP values were calculated by comparison with the calibration curve of Trolox and ferrous sulfate and are shown as mmol Trolox equivalent per gram of dry basis of seaweed (mM TE/g) and mmol ferrous sulfate per gram of dry basis of seaweed (mM Fe(II)E/g), respectively.

### 2.6 Statistical analysis

Data are expressed as means ± standard deviations and analyzed using one-way ANOVA and the following Duncan’s *post hoc* test at a 0.05 probability level in SPSS v. 26.0 (SPSS Inc. Chicago, IL, United States). Pearson’s correlation analysis was conducted using two-tailed tests in SPSS version 26.0. Principal component analysis (PCA), hierarchy process analysis (HCA), and a heatmap of the correlation were conducted using Origin v. 2021 software.

## 3 Results and discussions

### 3.1 Total phenolic content, TPhC, and TFC

**Figure 1:**
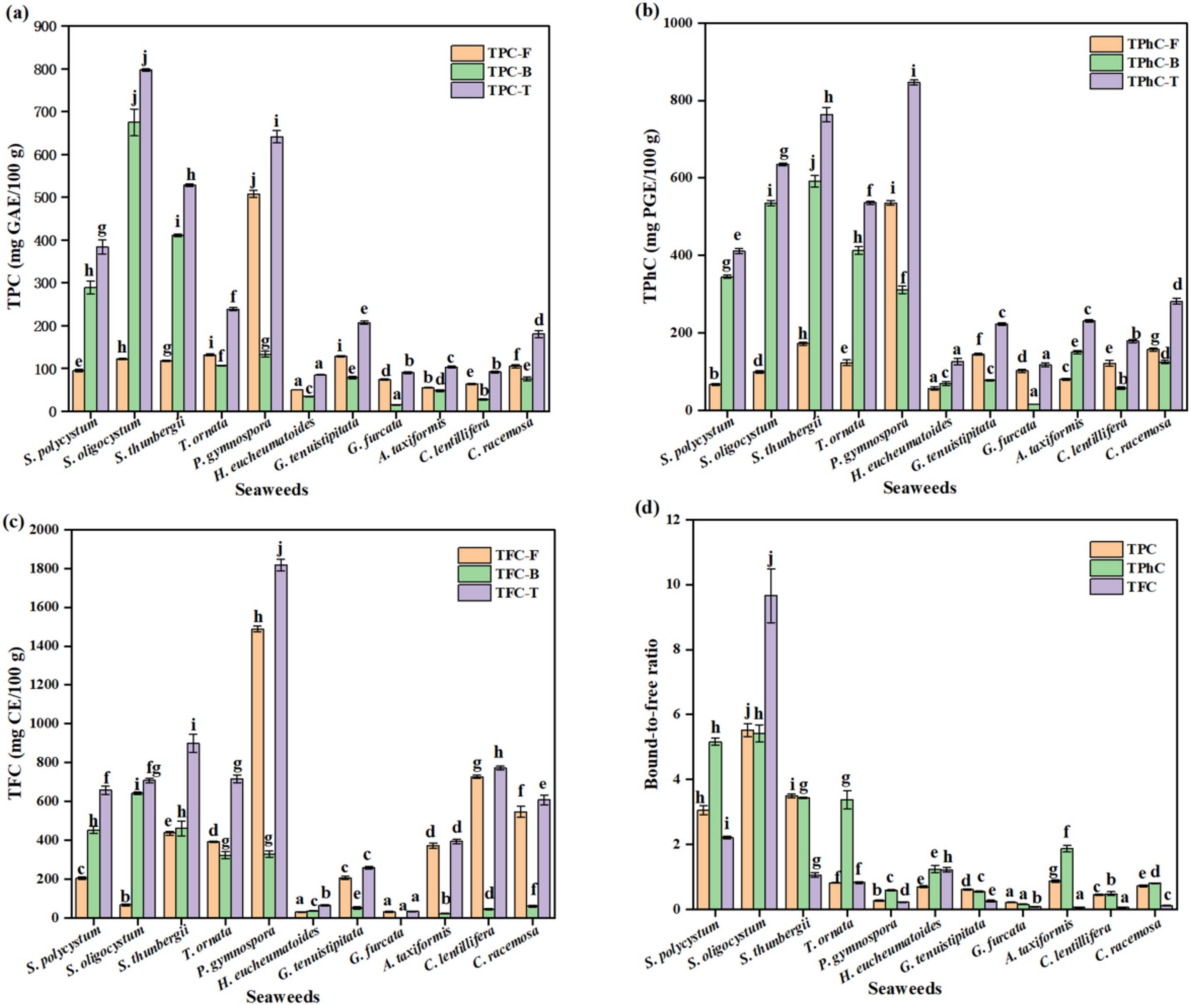
The free, bound, and total TPC **(a)**, TPhC **(b)**, and TFC **(c)**, and their bound-to-free ratio **(d)** of different seaweed species. Values with no common letters in each column are significantly different (*p* < 0.05). Each value represents the mean ± SD of three replicates. TPC is the abbreviation of total phenolic content, expressing the as mg GAE/100 g. GAE, gallic acid equivalent. TPhC is the abbreviation of total phlorotannin content, expressing the as mg PGE/100 g. PGE, phloroglucinol equivalent. TFC is the abbreviation of total flavonoid content, expressing the as mg CE/100 g. CE, catechin equivalent.

As shown in Figure 1, significant differences were observed in the free, bound, and total TPC, TPhC, and TFC among the different seaweed species (*p* < 0.05). The free, bound, and total TPC changed from 50.32 (*H. eucheumatoides*) to 507.41 (*P. gymnospora*), 15.55 (*G. furcata*) to 674.43 (*S. oligocystum*), and 85.25 (*H. eucheumatoides*) to 796.55 mg GAE/100 g (*S. oligocystum*), respectively. The highest free, bound, and total TPC were 10.08-fold, 43.37-fold, and 9.34-fold of the lowest values, respectively (*p* < 0.05). The free, bound, and total TPhC ranged from 56.50 (*H. eucheumatoides*) to 535.16 (*P. gymnospora*), from 15.49 (*G. furcata*) to 591.82 (*S. thunbergii*), and from 117.19 (*G. furcata*) to 846.49 mg PGE/100 g (*P. gymnospora*), respectively. *P. gymnospora* showed the highest free and total TPhC values, which were 9.47- and 7.22-fold higher, respectively, than the lowest values (*p* < 0.05). *S. thunbergii* showed the highest bound TPhC, which was 38.21-fold higher than that of *G. furcata* (*p* < 0.05). Moreover, the free, bound, and total TFC ranged from 30.84 (*G. furcata*) to 1488.19 mg CE/100 g (*P. gymnospora*), 2.43 (*G. furcata*) to 641.09 mg CE/100 g (*S. oligocystum*), and 33.27 (*G. furcata*) to 1817.57 mg CE/100 g (*P. gymnospora*), respectively. The highest free, bound, and total TFC were 48.26-fold, 263.82-fold, and 54.63-fold higher than the lowest values, respectively (*p* < 0.05). It is commonly believed that phenolics in seaweeds are synthesized via numerous metabolic pathways, contributing to the difference in existing forms, types, and content of phenolics (12). The differences in the free or bound TPC, TPhC, and TFC in previous studies were mainly attributed to harvesting location and period, genetic factors, and extraction method (1, 21, 30, 31). All 11 seaweed species used in this study were collected from locations with similar environmental conditions (Hainan Province) and harvested at respective maturity period. Thus, the observed discrepancies in free or bound TPC, TPhC, and TFC among seaweed species may be mainly attributed to genetic factors.

Several past studies have focused on free phenolics, whereas only sporadic studies on the bound form are available. Many previous studies have reported that the free TPC and TFC vary significantly among seaweed species (30, 32, 33). García-Casal et al. (32) found that free TPC and TFC in brown seaweeds were higher than those in red and green seaweeds, whereas Sapatinha et al. (30) found the opposite. In the present study, free TPC and TFC varied significantly among the seaweed species, which is in accordance with previous studies (30, 32). Although the highest free TPC and TFC were observed in brown seaweed *P. gymnospora*, brown seaweeds did not show an absolute predominance of free TPC and TFC compared to the other seaweeds. Moreover, significant differences in bound TPC were observed among the brown seaweeds, ranging from 779.00 to 1814.00 mg GAE/100 g (18). Our study also found a remarkable variation in bound TPC among different seaweeds. Previous studies showed bound TPC of *S. thunbergii* and *S. polycystum* were 1631.00 and 274.27 mg GAE/100 g, respectively (18, 21). The discrepancy between previous data and our results may be attributed to different extraction methods, harvest locations, and harvest periods affecting the accumulation of phenolic compounds (34). Importantly, the bound TFC and bound and total TPC in brown seaweeds were significantly higher than those in red and green seaweeds. Furthermore, in addition to phenolic acids, bromophenols, and flavonoids, phlorotannins are found in seaweeds, especially brown seaweeds (2). Petchidurai et al. (31) reported that free TPhC varied significantly among 32 seaweed species, showing 18.35-, 3.16-, and 4.13-fold differences among 11 brown, nine red, and 12 green seaweed species, respectively. Although the highest free TPhC was observed in the brown seaweed *Colpomenia sinuosa*, no significant differences were found in free TPhC among brown, red, and green seaweeds (31), which is consistent with our results. The brown seaweed *Fucus vesiculosus*, the species with the highest TPhC, had a free TPhC of 8,000 mg PGE/100 g in a study by Koivikko, which was significantly higher than the values observed in the present study (22). Koivikko et al. (22) also found that the bound TPhC of *Fucus vesiculosus* was 840.00 mg PGE/100 g, which was close to the bound TPhC of *S. thunbergii* observed in this study. The present study showed significantly higher levels of bound and total TPhCs in brown seaweed than in red and green seaweed.

Phenolics in seaweed and other plants are typically divided into free and bound forms (12). The ratio of bound to free phenolics (bound-to-free ratio) differs significantly among different plants (14, 15). This ratio was 0.07–3.20 in fruits (15) and 0.11–0.60 in vegetables (14). However, there is little available data on the ratio of seaweed. The bound-to-free ratio of TPC and TFC in *S. polycystum* reported by Wu et al. (21) were 3.98 and 2.42, respectively. The bound-to-free ratio of TPhC in the brown seaweed *Fucus vesiculosus* was 0.11 (22). In a previous study, free and bound phenolics were identified in the brown seaweed *Padina tetrastromatica*, but only free TPC was reported (24). In this study, significant differences in the bound-to-free ratios of TPC, TPhC, and TFC were observed (*p* < 0.05; Figure 1). The bound-to-free ratio of TPC, TPhC, and TFC varied from 0.21 to 5.52, from 0.15 to 5.42, and from 0.06 to 9.66, respectively, in which the highest values were found in *S. oligocystum.* The lowest bound-to-free ratios of TPC and TPhC were observed in *G. furcata*, whereas the lowest ratios of TFC were observed in *A. taxiformis* and *C. lentillifera.* In *S. polycystum*, *S. oligocystum*, and *S. thunbergii*, the bound-to-free ratios of TPC, TPhC, and TFC were greater than 1, indicating that bound phenolics were the major forms. The ratios of TPhC in *T. ornata*, *A. taxiformis*, *H. eucheumatoides* and this ratio of TFC in *H. eucheumatoides* were also greater than 1. The previously reported bound-to-free ratios of TPC and TFC in *S. polycystum* (3.98 and 2.41, respectively) were higher than those reported in this study (21). In a study by Wu et al. (15), oven-dried seaweed was extracted using the same solvent and method as those used for free and bound phenolics in the present study. When compared to the vacuum freeze-drying method, the oven-drying method promotes the degradation of free phenolics (35). Thus, the difference in the bound-to-free ratio of TPC and TFC in *S. polycystum* between the previous study and the present study may be due to the different drying methods.

### 3.2 Identification of free and bound phenolic compounds

Fifteen phenolic acids, including seven benzoic acid and its derivatives (peak 1–7) and eight hydroxycinnamic acid and its derivatives (peak 8–15), were identified, of which 11 and 13 compounds were present in free and bound forms, respectively (Table 1). Gallic acid, 2-hydroxybenzoic acid, syringic acid, and protocatechualdehyde were tentatively identified by a − 44 Th loss corresponding to the carboxylic acid group or − 29 Th loss corresponding to the aldehyde group (18, 36, 37). Vanillin displayed product ions at m/z 138.00, 125.00, and 93.04, corresponding to methyl group loss, CO loss, and methanol loss together with CO loss, respectively (38). Ethyl vanillin and ethyl gallate were tentatively identified by virtue of the vanillin (m/z 136.21) and gallic acid (m/z 168.91) fragments for the release of the ethyl moiety (36, 39). *p*-coumaric, ferulic, and *trans*-cinnamic acids showed the precursor ions [M − H]^−^ at m/z 163.10, 193.00, 146.95 and their product ions at m/z 119.01 [M–H–CO_2_]^−^, 91.01 [M–H–CO_2_–C_2_H_4_]^−^ (peak 8), 178.00 [M–H–CH_3_]^−^, 149.00 [M–H–CO_2_]^−^ (peak 9), and 118.94 [M − H − CO]^−^ (peak 10), respectively (18, 37). Caftaric acid, chlorogenic acid, rosmarinic acid, cynarin, and coutaric acid were identified by tartrate fragment (m/z 149.00) (peak 11), quinic acid fragment (m/z 191.00), caffeic acid fragment (m/z 179.2) (peak 12), dihydroxyphenyl-lactic acid fragment (m/z 196.96), a loss of H_2_O from caffeic acid fragment (m/z 161.00) (peak 13), caffeoylquinic acid fragment (m/z 352.94), quinic acid fragment (m/z 191.01) (peak 14), and tartrate fragment (m/z 149.10), coumaric acid fragment (m/z 163.11), a loss of CO_2_ from coumaric acid fragment (m/z 119.11) (peak 15), respectively (36, 40–42).

**Table 1:** The identification of phenolic compounds in free and bound fractions of 11 seaweed species by UHPLC-QQQ-MS.

Peak no.	λ_max_ (nm)	Tentative assignment	Model	Parent ions	ions	Reference
1	271	gallic acid	−	168.96	125.00, 97.00	(36)
2	260,294	2-hydroxybenzoic acid	−	137.00	93.00	(37)
3	275	syringic acid	+	199.00	155.10,140.10	(36)
4	274,308	protocatechualdehyde	−	136.90	108.00, 80.99	(18)
5	275	vanillin	+	153.00	138.00, 125.00, 93.04	(38)
6	280,310	ethyl vanillin	−	164.95	136.21, 92.05	(39)
7	272	ethyl gallate	−	196.94	168.91, 124.10	(36)
8	270,307	*p*-coumaric acid	−	163.10	119.01, 91.01	(36)
9	299,323	ferulic acid	−	193.00	178.00, 149.00, 134.00	(36)
10	278,306	trans-cinnamic acid	−	146.95	118.94, 77.01, 40.07	(37)
11	320	caftaric acid	−	311.00	179.00, 149.00, 135.00	(36)
12	295,327	chlorogenic acid	−	353.20	191.00, 179.20	(40)
13	290,328	rosmarinic acid	−	358.96	196.96, 161.00	(41)
14	242,304	cynarin	−	514.91	352.94,191.01	(42)
15	230,321	coutaric acid^a^	−	295.10	163.11,149.10,119.10	(36)
16	280	catechin	−	289.07	244.90, 204.90, 137.10	(43)
17	280	epicatechin	−	289.07	244.90, 204.90, 137.10	(43)
18	270	epigallocatechin	−	304.98	179.01, 124.98	(43)
19	270	gallocatechin	−	304.98	179.01, 124.98	(43)
20	274	epigallocatechin gallate	−	456.90	331.00, 169.04, 125.00	(43)
21	274	epicatechin gallate	−	440.89	288.99,168.98,124.97	(44)
22	274	gallocatechin gallate	−	456.90	331.00, 169.04, 125.00	(43)
23	280	procyanidin B1	−	576.89	288.99, 406.92, 424.93	(1)
24	280	procyanidin B2	−	576.89	288.99, 406.92	(1)
25	254,372	myricetin	−	317.00	178.95, 150.99	(40)
26	255,347	quercetin	−	301.01	178.99, 150.99	(37)
27	248,352	morin	−	300.83	106.97, 124.98, 150.99	(45)
28	257,356	quercitrin	−	447.00	301.00,179.00,151.00	(40)
29	256,354	guaiaverin	−	432.87	270.94, 300.94	(1)
30	266,348	kaempferol-3-O-rutinoside	−	592.91	254.91, 284.80	(1)
31	264,346	astragaline	−	446.90	285.06, 254.94, 226.98,	(1)
32	254,354	narcissin	−	624.87	478.88, 316.94, 85.05	(1)
33	288	taxifolin	−	302.83	284.98, 176.91, 124.98	(1)
34	292,330	aromadendrin	−	286.84	258.97, 124.98	(47)
35	286	taxifolin-7-O-rhamnoside	−	448.85	302.92, 284.91, 124.98	(1)
36	267,339	apigenin	+	271.01	227.02, 151.04	(40)
37	276	baicalein	+	271.01	122.90	(48)
38	267,345	diosmetin	+	301.00	153.00, 111.00, 255.00, 257.00	(49)
39	270,335	acacetin	+	285.01	153.00	(50)
40	272,330	hinokiflavone	−	537.00	417.00, 284.00	(21)
41	252, 348	cynaroside	−	446.89	284.78, 133.04, 107.03	(1)
42	270,350	isovitexin	−	431.01	280.91, 253.01	(51)
43	260,327	genistein	−	268.90	158.98, 132.90, 108.70	(40)
44	248	daidzein	−	253.10	208.00, 131.90, 91.10	(52)
45	283,327	hesperidin	−	608.95	301.02	(40)
46	287,327	naringenin	−	271.00	151.02, 107.02	(40)
47	280	leucocyanidin	+	306.92	126.99, 155.07	(53)
48	276,530	cyanidin	+	286.91	109.05, 137.01	(54)
49	274,327,503	keracyanin	+	594.87	448.92, 286.96, 136.99	(55)
50	516,323, 280	cyanidin-3-O-glucoside	+	448.86	286.93,137.01	(55)
51	280,306	*trans*-resveratrol	+	229.00	135.01, 107.11	(36)
52	282,308	*trans*-piceid	+	391.10	229.10, 135.00	(36)
53	280	4-bromophenol	−	172.07	80.86	(59)
54	280	2,4-dibromophenol	−	250.74	168.7, 81.37, 78.51	(59)
55	280	2,4,6-tribromophenol	−	330.60	81.35	(10, 56)
56	226,267	phloroglucinol	−	124.95	83.01,57.06, 41.05	(57)
57	280	diphlorethol/difucol^a^	−	249.00	231.00, 207.00,163.00, 113.00	(58)
58	280	eckol^a^	+	373.00	357.00, 319.00, 248.00, 231.00, 142.00	(21)
59	262,346	unknown	−	791.41	765.41, 575.02, 531.34, 461.50, 313.12, 277.23	
60	280	unknown	−	679.00	623.01, 578.01, 430.01, 340.01	
61	280	unknown	−	940.85	793.91, 686.84, 487.71,368.61, 249.41	

^a^Peaks were deduced by multiple reaction monitoring and comparison with the published data, without further recognization by comparison with respective commercial standards using UHPLC-QQQ-MS.

Thirty-five flavonoids, including nine flavan-3-ols (peak 16–24), eight flavonols (peak 25–32), three dihydroflavonols (peak 33–35), seven flavones (peak 36–42), two isoflavones (peak 43–44), two flavanones (peak 45–46), and four anthocyanidins (peak 47–51) were identified, of which 33 and 34 compounds were present in free and bound forms, respectively. Catechin and epicatechin were authenticated by virtue of CO_2_ loss (m/z 244.90) and retro Diel-Alder (RDA) fragmentation [(M − H – 152)^−^], along with different retention times (Table 1) (43). Peak 18–19 were authenticated as epigallocatechin and gallocatechin by virtue of a − 126 Th loss corresponding to the C_6_H_6_O_3_ group (m/z 179.01) and [C_6_H_6_O_3_ − H]^−^ ion (m/z 124.98), with different retention times. Epigallocatechin gallate, epicatechin gallate, gallocatechin gallate displayed [M − H − 126]^−^ ion, gallic acid fragment (m/z 169.04, 168.98, 169.04), and a loss of CO_2_ from gallic acid fragment (m/z 125.00, 124.97, 125.00), which were consistent with the previous studies (43, 44). Procyanidin B1 and B2, with different retention times, were authenticated by the fragments at m/z 288.99 [M − H]^−^ for the loss of the (epi) catechin entity (1). Myricetin, quercetin, and morin displayed the deprotonated ions [M − H]^−^ at m/z 317.00, 301.01, and 300.83, respectively, and typical RDA fragments (m/z 178.95, 178.99, 150.99) (37, 40, 45). Peaks 28–32 were identified as quercitrin, guaiaverin, kaempferol-3-O-rutinoside, astragaline, narcissin by the loss of rhamnoside (−146 Th), arabinoside (−132 Th), glucoside (−162 Th) or rutinoside (−308 Th) group (1, 42). Taxifolin and aromadendrin showed the parent ions at m/z 302.83 and 286.84, respectively, and product ions at m/z 284.98 [M − H − H_2_O]^−^, 176.91 [M − H − C_6_H_6_O_3_]^−^, 124.98 [C_6_H_6_O_3_ − H]^−^ (Peak 32), and 258.97 [M − H − CO]^−^, 124.98 [C_6_H_6_O_3_ − H]^−^ (Peak 33), which were previously reported (46, 47). Taxifolin-7-O-rhamnoside was identified by the taxifolin fragment (m/z 302.92), a loss of H_2_O from the taxifolin fragment (m/z 284.91), and [C_6_H_6_O_3_ − H]^−^ ion (m/z 124.98) (1). Apigenin displayed the parent ion [M + H]^+^ at m/z 271.01 and a product ion at m/z 227.02 due to CO_2_ loss, as previously reported (40). Baicalein was identified by the parent ion [M + H]^+^ at m/z 271.01 and product ion at m/z 122.90, in accordance with previously reported data (48). Diosmetin and acacetin were authenticated by the fragment at m/z 153.00, resulting from RDA cleavage of the C-ring for both, and the fragment at m/z 257.00 (loss of CO_2_) for diosmetin (49, 50). Peak 40 displayed the parent ion [M − H]^−^ at m/z 537.00 and the product fragment at m/z 284.00, consistent with mass spectra data of hinokiflavone (21). Cynaroside showed the luteolin fragment (m/z 284.78) for the release of glucoside group (−162 Th) (1). Isovitexin generated the deprotonated ion [M − H]^−^ at m/z 431.01 and the fragments at m/z 280.91 [M − H − 150]^−^ and 253.01 [M − H − 150 − CO]^−^ as previously reported (51). Genistein was assigned through the deprotonated ion [M − H]^−^ at m/z 289.9 and the fragment at m/z 132.90 as previously reported (40). Peak 44, showing the parent ion [M − H]^−^ at m/z 253.10 and a product ion at m/z 91.10, were identified as daidzein (52). Hesperidin was distinguished by the deprotonated ion [M − H]^−^ at m/z 608.95 and its hesperitin fragment (m/z 301.02) for a loss of glycoside (40). Peak 46 showed the parent ion [M − H]^−^ at m/z 271.00 and product ions at m/z 151.02 (typical RDA fragment) and 107.02 (the formation of a hydroxytropylium ion), which were in accordance with mass spectra data of naringenin (18). Four anthocyanidins (peak 47–50) were identified by the parent ions [M + H]^+^ at m/z 306.92, 286.91, 594.87, and 448.86 and their product ions corresponding to the fragments [M + H − 152]^+^ (m/z 155.07), [M + H − 152 − CO]^+^ (m/z 126.99) for leucocyanidin, [M + H − 150]^+^ (m/z 137.01), [M + H − 150 − CO]^+^ (m/z 109.01) for cyanidin, rutinoside loss for keracyanin, and glucoside loss for cyanidin-3-O-glucoside (53–55).

Peak 51 showed the parent ion [M + H]^+^ at m/z 229.00, and product ions at m/z 135.01 (phenol group loss) and m/z 107.11 (the formation of a hydroxytropylium ion formation), which were in accordance with the mass spectra data of *trans*-resveratrol (Table 1) (36). *Trans*-piceid was distinguished by the resveratrol fragment resulting from a loss of a glucoside group (−162 Th) and further loss of a phenol group (36).

Three bromophenols, with parent ions at m/z 172.07, 250.74, and 330.60, produced fragment ions at m/z 80.90 for 4-bromophenol, m/z 81.40, and 78.50, respectively, for 2,4-dibromophenol, and m/z 81.35 for 2,4,6-tribromophenol, as reported previously (10, 56).

Peak 56, displaying a deprotonated ion [M − H]^−^ at m/z 124.95 and a product ion at m/z 83.01 deriving from the loss of CO and CH_2_ together, was authenticated as phloroglucinol (57). Peak 57 displayed a deprotonated ion at m/z 249.00 and a product ion at m/z 231.00, resulting from H_2_O loss, which was consistent with the mass spectra data for diphlorethol and difucol reported in previous studies (21, 58). The dimers diphlorethol and difucol, which are composed of two phloroglucinol units through phenyl and ether bonds, respectively, cannot be distinguished by mass spectral data without authentic compounds. Peak 58 with the parent ion [M + H]^+^ at m/z 373.00 and the fragments [M + H − 125]^+^ (m/z 248.00) and [M + H − 16]^+^ (m/z 357.00) were observed, which corresponded to eckol (21).

Sporadic research has reported on the bound phenolic profiles of seaweeds. Bound phenolics, including gallic acid and coutaric acid, are abundant in *S. polycystum*. Bound flavonoids such as luteolin, acacetin derivative, baicalein, diosmetin, and apigenin were detected in *S. polycystum* and *Padina tetrastromatica* by LC–MS (21, 24). Myricetin, morin, hesperidin, quercitrin, cirsimaritin, rutin, and kaempferol in their bound forms were detected by HPLC-DAD in 27 green, red, and brown seaweeds (45). Koivikko et al. (22) found that the brown seaweed *Fucus vesiculosus* is rich in bound phlorotannins that were not previously identified (22). Although bound eckol is not found in seaweeds, dioxynohydroeckol derivatives have been identified in the bound fraction of *Padina tetrastromatica* using LC–MS (24). Forty-two phenolic compounds were identified in the bound fraction of seaweeds for the first time, although their free counterparts have also been found in seaweeds (1, 24, 59).

### 3.3 Quantification of free and bound phenolic compounds by UPLC-QQQ-MS

The amounts of phenolics identified in the free and bound seaweed fractions were determined by UPLC-QQQ-MS (Table 2). Significant diversity in the number and amount of phenolic compounds in free and bound forms was observed among seaweed species (*p* < 0.05), which is in accordance with previous studies (45, 60, 61). The free, bound, and total phenolic numbers ranged from 2 (*C. lentillifera*) to 38 (*P. gymnospora*), 6 (*S. polycystum*) to 33 (*T. ornata*), and 9 (*C. lentillifera*) to 65 (*P. gymnospora*), respectively. The free, bound and total phenolic amount were in the range of 130.97–11106.83, 3296.18–9534.50, and 4929.07–16743.00 μg/10 g, in which the highest were, respectively, shown in *T. ornata*, *G. tenuistipitata*, and *T. ornata*, whereas the lowest were, respectively, observed in *H. eucheumatoides*, *S. polycystum*, and *S. polycystum*.

**Table 2:** The amount of phenolic compounds in free and bound extracts of seaweeds determined by UHPLC-QQQ-MS.

	Form	Amount (μg/10 g)										
		*S. polycystum*	*S. oligocystum*	thunbergii	ornata	gymnospora	eucheumatoides	tenuistipitata	*G. furcata*	*A. taxiformis*	*C. lentillifera*	*C. racemosa*
gallic acid	B	97.92 ± 1.87^a^	nd	nd	nd	nd	nd	nd	nd	nd	nd	nd
2-hydroxybenzoic acid	F	nd	0.63 ± 0.03^c^	0.63 ± 0.05^c^	0.42 ± 0.04^ab^	3.43 ± 0.21^f^	0.35 ± 0.02^a^	2.24 ± 0.34^e^	0.91 ± 0.10^d^	0.77 ± 0.08^cd^	nd	0.49 ± 0.05^b^
	B	nd	0.50 ± 0.03^c^	nd	nd	nd	0.30 ± 0.02^b^	2.40 ± 0.31^e^	0.35 ± 0.03^b^	0.21 ± 0.01^a^	nd	1.30 ± 0.10^d^
syringic acid	B	nd	nd	23.10 ± 0.50^c^	19.20 ± 0.38^b^	16.20 ± 0.48^a^	nd	nd	nd	nd	nd	nd
protocatechualdehyde	F	nd	23.73 ± 1.00^c^	99.26 ± 2.30^e^	nd	9.87 ± 0.54^a^	nd	70.70 ± 1.23^d^	nd	nd	nd	18.83 ± 0.87^b^
	B	nd	183.40 ± 2.35^e^	nd	nd	72.20 ± 1.87^d^	4.90 ± 0.23^a^	nd	29.30 ± 0.58^c^	nd	nd	10.10 ± 0.54^b^
vanillin	F	nd	53.41 ± 0.51^d^	421.82 ± 5.41^h^	184.66 ± 4.23^f^	17.36 ± 0.84^b^	23.52 ± 0.25^c^	18.76 ± 0.75^b^	91.49 ± 1.89^e^	200.55 ± 2.35^g^	nd	9.03 ± 0.24^a^
	B	nd	19.12 ± 0.32^c^	59.15 ± 0.67^h^	40.70 ± 1.23^g^	30.69 ± 0.78^f^	13.41 ± 0.46^b^	32.42 ± 0.99^f^	8.82 ± 0.32^a^	22.01 ± 0.69^d^	nd	27.72 ± 0.44^e^
ethyl vanillin	F	nd	nd	0.63 ± 0.03^d^	0.35 ± 0.03^b^	0.49 ± 0.03^c^	nd	0.35 ± 0.02^b^	0.42 ± 0.04^bc^	0.21 ± 0.01^a^	nd	nd
	B	nd	0.40 ± 0.02^b^	nd	nd	nd	nd	0.60 ± 0.02^c^	0.21 ± 0.01^a^	0.20 ± 0.01^a^	nd	0.40 ± 0.03^b^
ethyl gallate	F	nd	0.07 ± 0.01^a^	nd	nd	0.14 ± 0.01^b^	nd	nd	nd	nd	nd	nd
*p*-coumaric acid	F	nd	nd	nd	nd	0.28 ± 0.02^a^	nd	nd	nd	1.40 ± 0.10^b^	nd	nd
	B	nd	nd	0.84 ± 0.02^d^	0.60 ± 0.03^c^	0.40 ± 0.02^b^	1.30 ± 0.10^e^	47.40 ± 0.87^g^	0.05 ± 0.01^a^	33.40 ± 0.58^f^	nd	nd
ferulic acid	B	nd	nd	nd	nd	nd	nd	300.20 ± 4.82^a^	nd	nd	nd	nd
*trans*-cinnamic acid	F	nd	nd	nd	1.05 ± 0.06^c^	1.54 ± 0.05^e^	nd	1.26 ± 0.10^d^	0.07 ± 0.01^a^	0.28 ± 0.02^b^	nd	1.12 ± 0.10^cd^
	B	nd	1.54 ± 0.04^d^	0.98 ± 0.05^c^	0.40 ± 0.02^b^	0.90 ± 0.06^c^	0.20 ± 0.01^a^	2.20 ± 0.05^e^	2.15 ± 0.04^e^	2.50 ± 0.11^f^	nd	1.60 ± 0.07^d^
caftaric acid	F	nd	nd	nd	0.70 ± 0.02^a^	0.84 ± 0.02^b^	nd	nd	nd	nd	nd	nd
	B	nd	2.90 ± 0.10^b^	6.71 ± 0.31^c^	9.20 ± 0.32^d^	2.20 ± 0.13^a^	3.15 ± 0.20^b^	nd	nd	nd	nd	nd
chlorogenic acid	F	55.67 ± 1.02^a^	nd	nd	nd	nd	nd	nd	nd	nd	nd	nd
rosmarinic acid	F	nd	nd	0.07 ± 0.01^a^	nd	nd	nd	nd	nd	0.07 ± 0.01^a^	nd	0.14 ± 0.01^b^
	B	nd	0.10 ± 0.01^b^	0.10 ± 0.01^b^	0.10 ± 0.01^b^	nd	0.11 ± 0.01^b^	nd	0.07 ± 0.01^a^	0.20 ± 0.01^c^	nd	0.11 ± 0.01^b^
cynarin	F	nd	0.60 ± 0.02^a^	1.10 ± 0.08^b^	nd	nd	nd	nd	nd	nd	nd	nd
	B	nd	nd	nd	0.10 ± 0.01^a^	nd	nd	nd	nd	nd	nd	nd
coutaric acid^#^	B	1800.08 ± 35.42^a^	nd	nd	nd	nd	nd	nd	nd	nd	nd	nd
phenolic acid number	F	1	5	6	5	8	2	5	4	6	0	5
	B	2	7	6	7	6	7	6	7	6	1	6
phenolic acid amount	F	55.67 ± 1.02^d^	78.44 ± 1.48^e^	523.54 ± 7.58^i^	187.18 ± 4.31^g^	33.95 ± 1.50^c^	23.87 ± 0.23^a^	93.31 ± 2.08^f^	92.89 ± 2.14^f^	203.28 ± 2.40^h^	nd	29.61 ± 1.02^b^
	B	1898.00 ± 37.29^j^	207.96 ± 2.54^h^	90.88 ± 1.21^e^	70.30 ± 1.85^d^	122.59 ± 2.78^f^	23.37 ± 0.81^a^	385.23 ± 5.54^i^	40.95 ± 0.85^b^	58.52 ± 1.18^c^	176.02 ± 2.35^g^	41.23 ± 0.87^b^
catechin	F	nd	nd	0.70 ± 0.04^b^	0.98 ± 0.04^d^	0.63 ± 0.03^b^	1.05 ± 0.05^de^	5.32 ± 0.25^f^	0.42 ± 0.02^a^	0.84 ± 0.02^c^	nd	1.19 ± 0.08^e^
	B	nd	nd	0.90 ± 0.03^b^	nd	12.40 ± 0.58^f^	1.30 ± 0.11^c^	nd	nd	8.30 ± 0.78^e^	5.30 ± 0.25^d^	0.21 ± 0.01^a^
epicatechin	F	nd	0.21 ± 0.01^c^	nd	nd	nd	0.07 ± 0.01^a^	0.07 ± 0.01^a^	nd	0.14 ± 0.01^b^	nd	0.14 ± 0.01^b^
	B	nd	7.30 ± 0.24^g^	4.10 ± 0.23^f^	4.02 ± 0.12^f^	0.70 ± 0.01^e^	0.30 ± 0.02^c^	0.50 ± 0.02^d^	0.15 ± 0.01^b^	0.70 ± 0.02^e^	nd	0.10 ± 0.01^a^
epigallocatechin	F	nd	0.56 ± 0.03^a^	nd	nd	7.28 ± 0.35^f^	5.11 ± 0.24^e^	1.12 ± 0.09^b^	2.59 ± 0.13^c^	2.59 ± 0.11^c^	nd	3.29 ± 0.23^d^
	B	nd	71.30 ± 0.98^f^	nd	8.70 ± 0.12^d^	33.60 ± 0.87^e^	8.20 ± 0.23^c^	nd	nd	7.05 ± 0.15^b^	nd	1.30 ± 0.07^a^
gallocatechin	F	nd	0.42 ± 0.02^b^	0.35 ± 0.02^a^	0.35 ± 0.01^a^	0.63 ± 0.05^c^	1.05 ± 0.05^d^	nd	nd	0.42 ± 0.02^b^	nd	0.35 ± 0.02^a^
	B	nd	4.10 ± 0.20^e^	4.80 ± 0.10^f^	6.60 ± 0.23^g^	nd	nd	1.01 ± 0.05^b^	1.60 ± 0.11^d^	0.70 ± 0.03^a^	nd	1.30 ± 0.07^c^
epigallocatechin gallate	F	nd	nd	nd	nd	nd	nd	nd	nd	0.07 ± 0.01^a^	nd	0.07 ± 0.01^a^
	B	nd	nd	nd	0.10 ± 0.01^a^	nd	nd	0.10 ± 0.01^a^	nd	nd	nd	nd
epicatechin gallate	F	nd	0.21 ± 0.02^b^	nd	nd	nd	nd	0.07 ± 0.01^a^	nd	nd	nd	nd
	B	nd	nd	0.10 ± 0.01^a^	0.11 ± 0.01^a^	0.10 ± 0.01^a^	nd	nd	nd	0.11 ± 0.01^a^	nd	nd
gallocatechin gallate	F	nd	nd	nd	nd	0.07 ± 0.01^a^	0.07 ± 0.01^a^	nd	nd	nd	nd	nd
	B	nd	0.20 ± 0.02^b^	nd	nd	nd	nd	0.10 ± 0.01^a^	nd	nd	nd	nd
procyanidin B1	F	nd	0.14 ± 0.01^a^	0.14 ± 0.01^a^	0.14 ± 0.01^a^	0.28 ± 0.02^c^	0.14 ± 0.01^a^	0.14 ± 0.01^a^	nd	0.21 ± 0.01^b^	nd	nd
	B	nd	0.31 ± 0.02^c^	nd	nd	nd	0.20 ± 0.01^b^	0.11 ± 0.01^a^	0.10 ± 0.01^a^	0.42 ± 0.02^d^	nd	0.20 ± 0.01^b^
procyanidin B2	F	nd	0.07 ± 0.01^a^	nd	nd	0.21 ± 0.01^c^	0.28 ± 0.02^d^	0.14 ± 0.01^b^	nd	0.14 ± 0.01^b^	nd	0.07 ± 0.01^a^
	B	nd	0.70 ± 0.04^c^	0.20 ± 0.01^a^	0.31 ± 0.02^b^	nd	0.21 ± 0.01^a^	nd	nd	0.20 ± 0.01^a^	nd	nd
myricetin	F	nd	nd	nd	7.91 ± 0.35^c^	3.50 ± 0.21^b^	nd	nd	nd	0.56 ± 0.02^a^	nd	nd
	B	nd	nd	2.40 ± 0.10^b^	11.10 ± 0.23^c^	nd	nd	388.40 ± 5.42^e^	22.85 ± 0.54^d^	nd	0.05 ± 0.01^a^	nd
quercetin	F	nd	nd	nd	0.21 ± 0.01^b^	nd	0.07 ± 0.01^a^	1.05 ± 0.08^e^	0.28 ± 0.01^c^	0.35 ± 0.02^d^	nd	0.21 ± 0.01^b^
	B	nd	0.14 ± 0.01^c^	3.50 ± 0.10^g^	nd	0.40 ± 0.03^e^	0.30 ± 0.02^d^	0.70 ± 0.04^f^	0.05 ± 0.01^a^	0.10 ± 0.01^b^	nd	nd
quercitrin	F	33.57 ± 1.31^c^	0.80 ± 0.04^b^	nd	0.07 ± 0.01^a^	0.07 ± 0.01^a^	nd	nd	nd	nd	nd	0.07 ± 0.01^a^
	B	nd	0.07 ± 0.01^a^	nd	nd	nd	nd	nd	nd	0.11 ± 0.01^b^	nd	nd
guaiaverin	F	nd	nd	nd	1.82 ± 0.10^c^	6.93 ± 0.25^d^	nd	0.42 ± 0.03^a^	nd	1.12 ± 0.05^b^	nd	nd
	B	nd	0.10 ± 0.01^a^	nd	nd	9.80 ± 0.43^b^	9.30 ± 0.34^b^	nd	nd	nd	nd	nd
morin	F	nd	nd	nd	2.52 ± 0.15^c^	0.14 ± 0.01^b^	0.07 ± 0.01^a^	nd	nd	nd	nd	0.14 ± 0.01^b^
	B	nd	0.30 ± 0.02^c^	0.10 ± 0.01^a^	0.20 ± 0.01^b^	0.30 ± 0.01^c^	1.60 ± 0.12^e^	373.40 ± 5.23^f^	1.05 ± 0.08^d^	nd	nd	nd
kaempferol 3-O-rutinoside	F	0.42 ± 0.03^b^	nd	nd	nd	0.07 ± 0.01^a^	nd	nd	nd	0.07 ± 0.01^a^	nd	nd
	B	nd	nd	0.10 ± 0.01^a^	0.11 ± 0.01^a^	nd	0.10 ± 0.01^a^	nd	nd	0.10 ± 0.01^a^	nd	nd
astragaline	F	nd	nd	0.07 ± 0.01^a^	nd	0.07 ± 0.01^a^	nd	nd	nd	nd	nd	nd
	B	nd	0.11 ± 0.01^a^	0.10 ± 0.01^a^	0.10 ± 0.01^a^	0.10 ± 0.01^a^	0.10 ± 0.01^a^	0.10 ± 0.01^a^	nd	0.10 ± 0.01^a^	nd	nd
narcissin	B	nd	nd	2.30 ± 0.07^a^	nd	nd	nd	nd	nd	nd	nd	nd
taxifolin	F	nd	0.14 ± 0.01^b^	nd	0.14 ± 0.01^b^	1.05 ± 0.05^d^	nd	nd	0.42 ± 0.04^c^	nd	nd	0.07 ± 0.01^a^
	B	nd	58.90 ± 1.25^e^	3.02 ± 0.25^c^	22.50 ± 0.23^d^	298.10 ± 1.23^f^	0.40 ± 0.02^b^	0.40 ± 0.03^b^	0.35 ± 0.02^b^	nd	nd	0.20 ± 0.01^a^
aromadendrin	F	nd	nd	0.42 ± 0.02^c^	0.21 ± 0.01^b^	0.14 ± 0.01^a^	nd	nd	nd	0.21 ± 0.01^b^	nd	nd
	B	nd	nd	0.10 ± 0.01^a^	0.11 ± 0.01^a^	nd	nd	0.10 ± 0.01^a^	nd	0.10 ± 0.01^a^	nd	nd
taxifolin 7-O-rhamnoside	F	nd	nd	nd	nd	nd	nd	nd	0.07 ± 0.01^a^	nd	nd	nd
baicalein	F	180.54 ± 5.23^g^	nd	13.37 ± 0.52^f^	nd	6.30 ± 0.21^c^	3.43 ± 0.12^b^	7.84 ± 0.21^d^	12.04 ± 0.23^e^	1.96 ± 0.08^a^	nd	8.19 ± 0.28^d^
	B	212.69 ± 7.85^f^	4.90 ± 0.12^a^	nd	15.40 ± 0.57^e^	nd	nd	9.30 ± 0.22^c^	5.50 ± 0.25^b^	13.50 ± 0.22^d^	nd	9.20 ± 0.34^c^
apigenin	F	nd	nd	0.07 ± 0.01^a^	0.14 ± 0.01^b^	nd	0.21 ± 0.01^c^	0.21 ± 0.01^c^	nd	0.07 ± 0.01^a^	nd	0.07 ± 0.01^a^
	B	165.65 ± 4.58^c^	0.20 ± 0.02^b^	nd	0.10 ± 0.01^a^	0.10 ± 0.01^a^	nd	0.11 ± 0.01^a^	nd	0.10 ± 0.01^a^	nd	nd
acacetin	F	19.82 ± 0.58^i^	0.56 ± 0.03^d^	1.54 ± 0.15^g^	0.14 ± 0.01^a^	4.34 ± 0.32^h^	0.42 ± 0.02^c^	0.98 ± 0.04^e^	0.21 ± 0.02^b^	1.19 ± 0.10^f^	nd	1.26 ± 0.07^f^
	B	nd	0.20 ± 0.02^b^	0.90 ± 0.04^d^	nd	0.11 ± 0.01^a^	nd	2.01 ± 0.15^e^	9.35 ± 0.35^f^	0.31 ± 0.02^c^	nd	0.82 ± 0.04^d^
diosmetin	F	465.87 ± 9.23^f^	nd	0.14 ± 0.01^a^	0.28 ± 0.01^b^	0.35 ± 0.02^c^	nd	nd	nd	0.71 ± 0.04^e^	nd	0.42 ± 0.02^d^
	B	820.54 ± 8.75^c^	nd	0.20 ± 0.01^a^	0.21 ± 0.01^a^	0.31 ± 0.02^b^	nd	0.30 ± 0.02^b^	nd	nd	nd	0.21 ± 0.01^a^
hinokiflavone	F	480.24 ± 10.23^c^	nd	1.89 ± 0.17^a^	2.24 ± 0.21^a^	5.32 ± 0.25^b^	nd	nd	nd	nd	3461.08 ± 32.21^d^	nd
	B	nd	61.31 ± 2.30^f^	nd	nd	0.10 ± 0.01^a^	nd	16.60 ± 0.58^c^	6.60 ± 0.33^b^	32.21 ± 1.50^e^	7129.90 ± 92.35^g^	26.70 ± 0.82^d^
cynaroside	F	nd	nd	0.14 ± 0.01^a^	nd	0.14 ± 0.01^a^	nd	nd	nd	nd	nd	nd
	B	nd	0.10 ± 0.01^a^	nd	0.20 ± 0.01^b^	nd	nd	nd	nd	0.11 ± 0.01^a^	nd	nd
isovitexin	F	nd	nd	0.14 ± 0.01^b^	nd	nd	0.07 ± 0.01^a^	0.14 ± 0.01^b^	nd	nd	nd	nd
	B	nd	nd	0.20 ± 0.01^b^	0.20 ± 0.01^b^	0.30 ± 0.02^c^	0.21 ± 0.01^b^	nd	0.05 ± 0.01^a^	nd	2.85 ± 0.12^d^	nd
genistein	B	nd	32.57 ± 1.02^b^	nd	0.11 ± 0.01^a^	nd	nd	nd	nd	nd	nd	nd
daidzein	F	nd	nd	2.23 ± 0.20^a^	nd	78.25 ± 2.31^b^	nd	nd	nd	nd	nd	nd
	B	nd	2.58 ± 0.12^a^	nd	nd	nd	nd	nd	nd	nd	nd	nd
hesperidin	F	nd	0.07 ± 0.01^a^	0.07 ± 0.01^a^	0.42 ± 0.02^d^	1.05 ± 0.07^e^	nd	0.07 ± 0.01^a^	0.07 ± 0.01^a^	0.28 ± 0.01^b^	nd	0.35 ± 0.02^c^
	B	nd	4.01 ± 0.25^d^	2.01 ± 0.15^c^	0.80 ± 0.05^a^	1.91 ± 0.13^c^	nd	1.40 ± 0.11^b^	6.75 ± 0.35^e^	3.60 ± 0.27^d^	nd	nd
naringenin	F	nd	nd	nd	nd	nd	nd	5.95 ± 0.25^a^	nd	nd	nd	nd
	B	nd	nd	nd	nd	nd	nd	3.91 ± 0.23^a^	nd	nd	nd	nd
leucocyanidin	F	nd	0.14 ± 0.01^a^	nd	nd	0.14 ± 0.01^a^	nd	nd	nd	nd	nd	nd
	B	nd	nd	nd	0.11 ± 0.01^a^	nd	0.10 ± 0.01^a^	0.10 ± 0.01^a^	nd	nd	nd	0.25 ± 0.01^b^
cyanidin	F	274.19 ± 1.25^c^	2215.85 ± 20.21^g^	1977.64 ± 28.23^f^	10846.64 ± 45.58^k^	1454.74 ± 35.20^d^	4.62 ± 0.21^a^	1803.06 ± 74.24^e^	4201.89 ± 55.24^h^	6089.23 ± 42.35^j^	83.51 ± 1.25^b^	4962.30 ± 28.57^i^
	B	199.30 ± 1.51^b^	4817.20 ± 22.58^f^	3377.90 ± 24.18^c^	5230.01 ± 54.21^g^	3787.90 ± 38.57^d^	6105.05 ± 55.87^h^	6353.20 ± 23.85^i^	4138.2 ± 47.82^e^	6977.02 ± 54.21^j^	32.25 ± 0.98^a^	3706.90 ± 41.32^d^
keracyanin	F	nd	0.07 ± 0.01^a^	nd	nd	nd	nd	nd	nd	nd	nd	nd
	B	nd	nd	nd	0.60 ± 0.02^b^	nd	nd	nd	nd	0.10 ± 0.01^a^	nd	0.11 ± 0.01^a^
cyanidin-3-O-glucoside	F	nd	nd	nd	nd	5.21 ± 0.24^a^	nd	nd	nd	nd	nd	nd
	B	nd	nd	0.11 ± 0.01^a^	nd	nd	nd	nd	nd	nd	nd	0.10 ± 0.01^a^
flavonoid number	F	7	13	15	16	24	14	15	9	18	2	15
	B	4	20	19	22	16	14	20	13	20	5	14
flavonoid amount	F	1454.65 ± 25.78^b^	2219.24 ± 20.01^f^	1998.911 ± 28.19^e^	10846.64 ± 45.37^k^	1576.911 ± 36.33^c^	16.66 ± 0.58^a^	1826.58 ± 74.88^d^	4217.99 ± 55.45^h^	6100.161 ± 42.26^j^	3544.59 ± 33.46^g^	4976.93 ± 28.12^i^
	B	1398.18 ± 21.32^a^	5066.4 ± 24.45^e^	3403.031 ± 24.01^b^	5301.701 ± 54.02^f^	4146.231 ± 38.87^d^	6127.36 ± 55.98^g^	7151.84 ± 24.43^i^	4192.6 ± 48.41^d^	7044.941 ± 55.69^h^	7170.35 ± 93.57^hi^	3747.50 ± 41.58^c^
*trans*-resveratrol	F	nd	0.28 ± 0.02^b^	0.28 ± 0.01^b^	0.28 ± 0.01^b^	0.98 ± 0.04^d^	nd	0.42 ± 0.03^c^	nd	0.21 ± 0.01^a^	nd	2.03 ± 0.15^e^
	B	nd	0.61 ± 0.02^d^	0.70 ± 0.03^e^	nd	0.64 ± 0.03^de^	nd	nd	0.05 ± 0.01^a^	0.41 ± 0.02^c^	nd	0.20 ± 0.01^b^
*trans*-piceid	F	nd	0.58 ± 0.03^a^	nd	nd	2.13 ± 0.08^b^	nd	nd	nd	nd	nd	nd
	B	nd	nd	0.10 ± 0.01^a^	0.11 ± 0.01^a^	0.10 ± 0.01^a^	nd	0.32 ± 0.02^b^	nd	nd	nd	nd
stilbene number	F	0	2	1	1	2	0	1	0	1	0	1
	B	0	1	2	1	2	0	1	1	1	0	1
stilbene amount	F	nd	0.86 ± 0.05^d^	0.28 ± 0.01^b^	0.28 ± 0.01^b^	3.11 ± 0.12^f^	nd	0.42 ± 0.03^c^	nd	0.21 ± 0.01^a^	nd	2.03 ± 0.15^e^
	B	nd	0.61 ± 0.02^f^	0.80 ± 0.04^g^	0.11 ± 0.01^b^	0.74 ± 0.04^g^	nd	0.32 ± 0.02^d^	0.05 ± 0.01^a^	0.41 ± 0.02^e^	nd	0.20 ± 0.01^c^
4-bromophenol	F	nd	nd	nd	nd	nd	nd	nd	nd	13.23 ± 0.23^a^	nd	nd
	B	nd	nd	1.22 ± 0.08^b^	nd	nd	nd	nd	nd	0.60 ± 0.03^a^	nd	nd
2,4-dibromophenol	F	1.96 ± 0.14^a^	32.27 ± 0.18^f^	18.41 ± 0.35^e^	67.83 ± 1.23^h^	11.90 ± 0.28^d^	89.46 ± 1.10^i^	10.15 ± 0.32^c^	6.44 ± 0.25^b^	1955.10 ± 21.25^j^	nd	50.89 ± 0.81^g^
	B	nd	59.10 ± 1.54^d^	30.40 ± 1.23^c^	18.40 ± 0.57^b^	32.00 ± 1.52^c^	81.70 ± 0.87^f^	64.61 ± 1.87^e^	nd	nd	18.01 ± 0.51^b^	6.50 ± 0.23^a^
2,4,6-tribromophenol	F	nd	0.77 ± 0.04^a^	3.50 ± 0.20^c^	4.90 ± 0.21^d^	3.85 ± 0.25^c^	0.98 ± 0.05^b^	5.67 ± 0.23^e^	nd	nd	nd	24.50 ± 0.40^f^
	B	nd	nd	nd	nd	nd	1.60 ± 0.06^a^	nd	nd	2.61 ± 0.18^b^	nd	nd
bromophenol number	F	1	2	2	2	2	2	2	1	2	0	2
	B	0	1	2	1	1	2	1	0	2	1	1
bromophenol amount	F	1.96 ± 0.14^a^	33.04 ± 0.22^e^	21.91 ± 0.55^d^	72.73 ± 1.44^f^	15.75 ± 0.53^c^	90.44 ± 1.15^g^	15.82 ± 0.55^c^	6.44 ± 0.25^b^	1968.33 ± 21.48^h^	nd	75.39 ± 1.21^f^
	B	nd	59.10 ± 1.54^e^	31.62 ± 1.15^d^	18.40 ± 0.57^c^	32.00 ± 1.52^d^	83.30 ± 0.93^g^	64.61 ± 1.87^f^	nd	3.21 ± 0.21^a^	18.01 ± 0.51^c^	6.50 ± 0.23^b^
phloroglucinol	F	nd	8.89 ± 0.23^b^	10.50 ± 0.34^c^	nd	6.58 ± 0.38^a^	nd	nd	nd	nd	nd	nd
	B	nd	nd	0.20 ± 0.01^a^	nd	nd	nd	1932.50 ± 24.35^b^	nd	nd	nd	nd
diphlorethol/difucol^#^	F	119.76 ± 1.87^c^	87.52 ± 1.44^b^	0.50 ± 0.03^a^	nd	158.25 ± 2.38^d^	nd	nd	nd	nd	nd	nd
	B	nd	125.23 ± 2.32^a^	287.23 ± 12.34^d^	245.12 ± 4.58^c^	200.21 ± 1.25^b^	nd	nd	nd	nd	nd	nd
eckol^#^	F	0.85 ± 0.02^a^	5.23 ± 0.22^b^	nd	nd	nd	nd	nd	nd	nd	nd	nd
	B	nd	1.23 ± 0.10^b^	20.35 ± 0.53^d^	0.54 ± 0.02^a^	2.23 ± 0.10^c^	nd	nd	nd	nd	nd	nd
phlorotannin number	F	2	3	2	0	2	0	0	0	0	0	0
	B	0	2	3	2	2	0	1	0	0	0	0
phlorotannin amount	F	120.61 ± 1.89^c^	101.64 ± 1.45^b^	11.00 ± 0.37^a^	nd	164.83 ± 2.76^d^	nd	nd	nd	nd	nd	nd
	B	nd	126.46 ± 2.22^a^	307.78 ± 12.86^d^	245.66 ± 4.56^c^	202.44 ± 1.33^b^	nd	1932.50 ± 24.35^e^	nd	nd	nd	nd
phenolic number	F	11	25	26	24	38	18	23	14	27	2	23
	B	6	31	32	33	27	23	29	21	29	7	22
	T	17	56	58	57	65	41	52	35	56	9	45
phenolic amount	F	1632.89 ± 28.82^b^ (33.13)*	2433.22 ± 23.41^e^ (30.82)	2555.64 ± 36.68^f^ (40.00)	11106.83 ± 51.13^k^ (66.34)	1794.55 ± 40.70^c^ (28.49)	130.97 ± 1.96^a^ (2.06)	1936.13 ± 77.54^d^ (16.88)	4317.32 ± 57.84^h^ (50.49)	8271.98 ± 66.15^j^ (53.79)	3544.59 ± 33.46^g^ (32.49)	5083.96 ± 30.50^i^ (57.26)
	B	3296.18 ± 58.61^a^ (66.87)	5460.53 ± 30.90^e^ (69.18)	3833.09 ± 39.28^b^ (60.00)	5636.17 ± 60.97^f^ (33.66)	4504.00 ± 44.48^d^ (71.51)	6234.03 ± 57.72^g^ (97.94)	9534.50 ± 55.49^j^ (83.12)	4233.6 ± 49.27^c^ (49.51)	7107.08 ± 57.10^h^ (46.21)	7364.38 ± 96.43^i^ (67.51)	3795.43 ± 42.69^b^ (42.74)
	T	4929.07 ± 87.43^a^	7893.75 ± 54.31^c^	6388.73 ± 75.96^b^	16743.00 ± 112.10^i^	6298.55 ± 85.18^b^	6365.00 ± 59.68^b^	11470.63 ± 133.03^g^	8550.92 ± 107.11^d^	15379.06 ± 123.25^h^	10908.97 ± 129.89^f^	8879.39 ± 73.19^e^

^#^The contents of coutaric acid, diphlorethol/difucol and eckol were quantified with the standard curve of gallic acid, phloroglucinol and phloroglucinol, respectively. The others were quantified by UHPLC-QQQ-MS with respective standard curves. *In forms, F and B represented free and bound forms. T represented total, namely the sum of free and bound. The contents were expressed as means ± SD (*n* = 3). The nd indicates not detected. Values not sharing a common letter within the same row are significantly different (*p* < 0.05).

In *S. polycystum*, 11 free and 6 bound phenolic compounds were detected, among which bound coutaric acid, bound and free diosmetin, free hinokiflavone, free and bound cyanidin, free and bound baicalein, bound apigenin, free diphlorethol/difucol, bound chlorogenic acid, and bound gallic acid accounted for 36.53, 16.64, 9.43, 9.74, 5.56, 4.04, 3.65, 4.30, 3.36, and 2.43% of total phenolic content, respectively. Among the 25 free and 31 bound phenolic compounds detected in *S. oligocystum*, cyanidin and diphlorethol/difucol in both free and bound form were the main components, accounting for 28.07, 61.03, 1.11, and 1.59% of total phenolic content, respectively. Among the 26 free and 32 bound phenolic compounds in *S. thunbergii*, bound and free cyanidin, free vanillin, free protocatechualdehyde, and bound diphlorethol/difucol were predominant, achieving 52.86, 30.95, 6.60, 1.55, and 4.50% of total phenolic content, respectively. The main compounds in *T. ornata* were free and bound cyanidin, bound diphlorethol/difucol, and free vanillin, achieving 64.78, 31.24, 1.46, and 1.10% of the total phenolic amount, respectively. *P. gymnospora* afforded high amounts of bound (60.14%) and free (23.10%) cyanidin, bound taxifolin (4.76%), free (2.51%) and bound diphlorethol/difucol (3.18%), bound protocatechualdehyde (1.15%), and free daidzein (1.24%). In *H. eucheumatoides*, bound cyanidin and free and bound 2,4-dibromophenol, respectively, achieved 95.92, 1.41, and 1.28% of total phenolic amount. In *G. tenuistipitata*, free and bound cyanidin, bound phloroglucinol, bound myricetin, and bound morin, respectively, achieved 15.72, 55.39,16.85, 3.39, and 3.26% of total phenolic content, respectively. In *G. furcata*, free and bound cyanidin and free vanillin accounted for 49.14, 48.39, and 1.07% of the total phenolic amount, respectively. In *A. taxiformis*, bound and free cyanidin, free 2,4-dibromophenol, and free vanillin accounted for 45.37, 39.59, 12.71, and 1.30% of total phenolic content, respectively. Among two green seaweeds, *C. lentillifera* was rich in free (31.73%) and bound hinokiflavone (65.36%), whereas free (55.89%) and bound cyanidins (41.75%) were dominant in *C. racemosa.* Among all phenolic compounds detected, cyanidin amount was the highest, ranging from 5242.64–16076.65 μg/10 g and accounting for 71.11–97.63% of the total phenolic amount in all seaweeds except *S. polycystum* and *C. lentillifera*.

In 29 brown, red, and green seaweeds, flavonoids were the predominant components, whereas phenolic acids were present in small amounts (45). Abirami and Kowsalya (62) did not observe flavonoids in the green seaweed *Ulva lactuca* or the red seaweed *Kappaphycus alvarezii*. Koivikko et al. (22) found that phlorotannins are dominant in the brown alga *Fucus vesiculosus*, of which free phlorotannins are the main components (22). A limited quantity of bromophenols has been found in seaweeds, resulting in relatively lower isolation and bioactive characteristics than those of phlorotannins (2). Among free phenolics, flavonoids achieved 78.22–97.66% of free phenolic content in brown seaweeds, 12.72% in *H. eucheumatoides* while 73.74–97.70% in other red seaweeds, and 97.89–100.00% in green seaweeds. Free phenolic acids accounted for 1.69–20.49% in brown seaweeds, 2.15–18.23% in red seaweeds, and 0.00–0.58% in green seaweeds of free phenolic amount. Free phlorotannins were found in four brown seaweeds, which accounted for 0.43–9.19% of free phenolic amount. In bound phenolics, phenolic acids accounted for 57.58% of bound phenolic amount in *S. polycystum* while 1.25–3.81% in other brown seaweeds, 0.37–4.04% in red seaweeds, and 1.09–2.39% in green seaweeds. Bound flavonoids achieved 42.42% of the bound phenolic content in *S. polycystum*, 88.76–94.07% in other brown seaweeds, 75.01–99.03% in red seaweeds, and 97.37–98.74% in green seaweeds. When considering the ratio of total flavonoid content to the total phenolic content, flavonoids accounted for 57.88% in *S. polycystum* and 78.27–98.36% in other seaweeds. These results indicated that flavonoids are the primary components of seaweeds, consistent with the results of Yoshie-Stark et al. (45). The diversity and amounts of seaweed metabolites, including phenolics, have been attributed to both abiotic and biotic factors, including species, life stage, location, light intensity, nutrient conditions, and extraction methods (2). Phlorotannins have been reported to act as chemical defenses against herbivory, and their amounts are consequently influenced by herbivory intensity (22). In *A. taxiformis*, the ratio of free phenolic acids, flavonoids, and bromophenols to the free phenolic content varies considerably with different solvents coupled with ultrasound, ranging from 0.27–20.26%, 68.37–99.64%, and 0.09–16.36%, respectively (59). These ratios, shown by Gao et al. (59) study by extraction with 70% ethanol coupled with ultrasound for 1 time, were 1.62, 82.01, and 16.36%, which were close to the ratios from the present study extracted by 70% ethanol coupled with homogenization for 2 times. Wu et al. (21) detected 11 free and 5 bound phenolic compounds from oven-dried *S. polycystum*, in which the ratio of free phenolic acids, flavonoids, and phlorotannins amount to free phenolic amount were 4.28, 84.97, 9.29%, this was significantly different with the results of the present study. The discrepancy of *S. polycystum* between previous data and the present results may be attributed to the different drying methods used for *S. polycystum* (34).

### 3.4 Antioxidant activity

**Figure 2:**
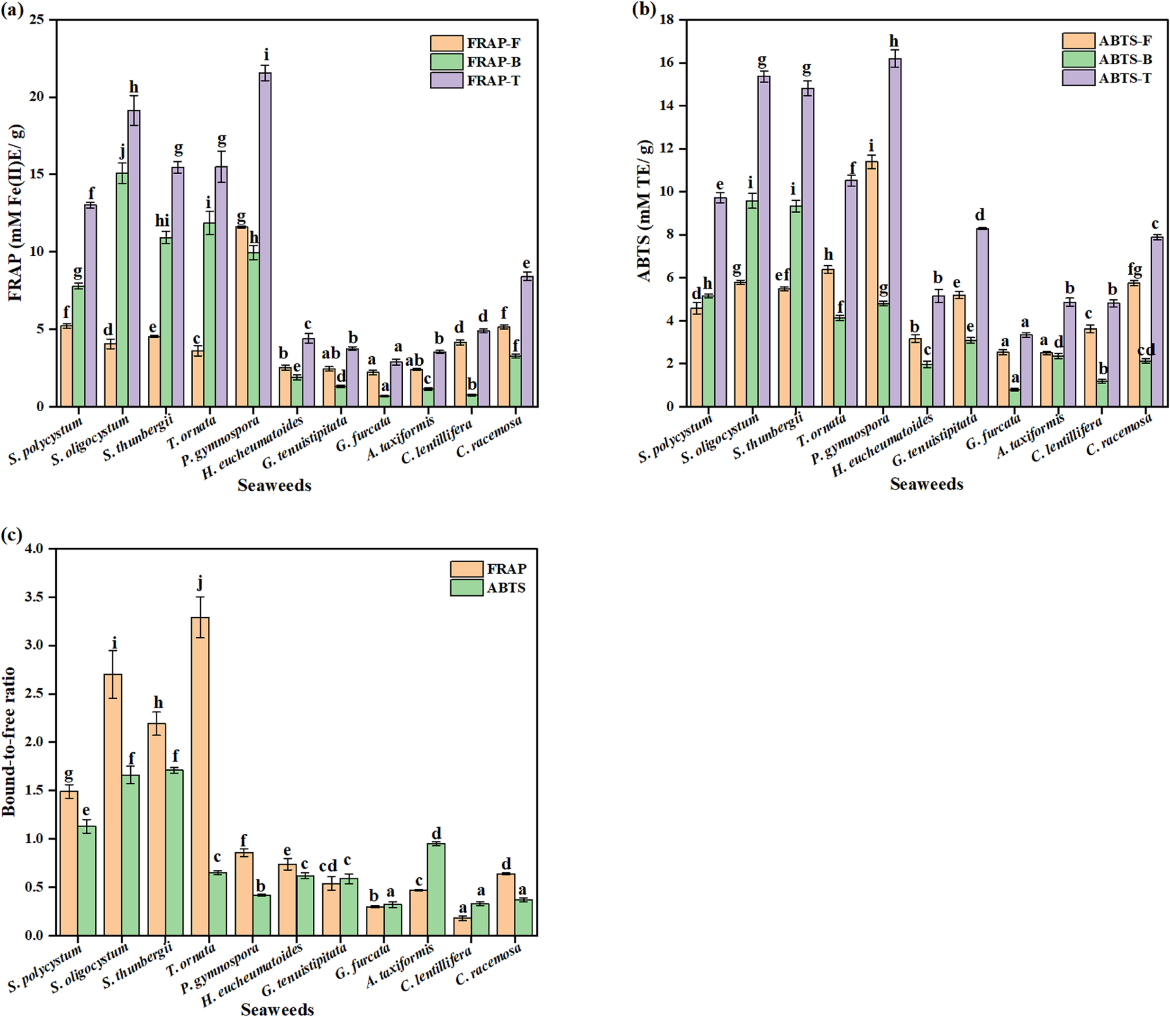
The free, bound, and total FRAP **(a)** and ABTS **(b)** values, and their bound-to-free ratio **(c)** of different seaweed species.Values with no common letters in each column are significantly different (*p* < 0.05). Each value represents the mean ± SD of three replicates.

The antioxidant activities of the free and bound seaweed fractions were evaluated using the FRAP and ABTS assays (Figure 2). Among the seaweed species, there were significant diversities found in the FRAP and ABTS values of the free and bound fractions, varying, respectively, in the range of 2.22–11.61 mM Fe(II)E/g, 0.67–15.08 mM Fe(II)E/g, 2.54–11.39 mM TE/g, and 0.80–9.58 mM TE/g (*p* < 0.05). *P. gymnospora* showed the highest free and total FRAP, and the second highest bound FRAP. The highest bound FRAP was observed in *S. oligocystum*. For ABTS, the highest free and total values were observed for *P. gymnospora*, and the highest bound values were observed for *S. oligocystum* and *S. thunbergii*. The lowest free FRAP was observed in *G. furcata*, *G. tenuistipitata* and *A. taxiformis*, and the lowest free ABTS was observed in *G. furcata* and *A. taxiformis*, and the lowest bound and total FRAP and ABTS values were observed in *G. furcata.* The bound-to-free ratios of FRAP and ABTS varied significantly among the seaweed species. In *S. polycystum*, *S. oligocystum*, and *S. thunbergii*, the bound-to-free ratios of FRAP and ABTS values were greater than 1, indicating the major contribution of bound phenolics to antioxidant activity. The bound-to-free ratio of FRAP was also greater than 1 for *T. ornata. The* free extracts of various seaweeds, including *A. taxiformis*, *S. polycystum*, and *Padina tetrastromatica*, have been demonstrated to possess antioxidant activities *in vitro* and *in vivo* (21, 24, 59, 60, 63). Potent antioxidant activity was also observed in the bound extracts of *S. polycystum* (21). Moreover, the bound-to-free ratio of antioxidant activity measured by the three assays was greater than 1 in *S. polycystum*, indicating that bound phenolics mainly contribute to the antioxidant activity (59).

### 3.5 Correlation among TPC, TPhC, TFC and antioxidant capacity

In the free fraction of seaweeds, a positive correlation was shown among antioxidant capacity (free FRAP and ABTS) and free TPC, TPhC and TFC, as indicated by the correlation between free FRAP and free TPC, TPhC and TFC (Pearson’s r = 0.918, 0.909, and 0.876, respectively, *p* ≤ 0.001), and the correlation between free ABTS and free TPC, TPhC and TFC [Pearson’s r = 0.929, 0.891 (*p* ≤ 0.001), 0.769 (*p* ≤ 0.01), respectively] (Table 3). Similarly, in the bound fraction of seaweeds, there was a positive correlation between the antioxidant capacity (bound FRAP and ABTS) and bound TPC, TPhC, and TFC (Table 3). A positive correlation was also observed between antioxidant capacity (total FRAP and ABTS) and TPC, TPhC, and TFC. These results revealed a positive correlation between phenolic content and antioxidant capacity, which is consistent with previous studies (21, 64). Bound TPC, TPhC, and TFC were positively correlated with antioxidant capacity (total FRAP and ABTS), whereas only free TPC was positively correlated with antioxidant capacity (total FRAP and ABTS) (*p* < 0.05), indicating that bound phenolics may be the primary contributors to antioxidant capacity.

**Table 3:** Pearson correlation coefficients among the free, bound, and total TPC, TPhC, TFC, and antioxidant activities.

	TPC			TPhC			TFC			FRAP			ABTS			
	Free	Bound	Total	Free	Bound	Total	Free	Bound	Total	Free	Bound	Total	Free	Bound	Total	
TPC	Free	1														
	Bound	0.057	1													
	Total	0.561	0.858***	1												
TPhC	Free	0.974***	−0.038	0.469	1											
	Bound	0.247	0.837**	0.820**	0.166	1										
	Total	0.687*	0.635*	0.879***	0.637*	0.866***	1									
TFC	Free	0.828**	−0.177	0.279	0.896***	0.085	0.521	1								
	Bound	0.289	0.907***	0.900***	0.166	0.936***	0.816**	0.049	1							
	Total	0.849***	0.269	0.659*	0.850***	0.509	0.829**	0.885***	0.508	1						
FRAP	Free	0.918**	0.133	0.582	0.909***	0.311	0.704*	0.876***	0.375	0.930***	1					
	Bound	0.397	0.804**	0.870***	0.282	0.941***	0.878***	0.156	0.945***	0.575	0.428	1				
	Total	0.659*	0.673*	0.897***	0.566	0.848***	0.950***	0.456	0.876***	0.801**	0.714*	0.938***	1			
ABTS	Free	0.929**	0.228	0.667*	0.891***	0.446	0.801**	0.769**	0.467	0.881***	0.894***	0.597	0.805**	1		
	Bound	0.219	0.936***	0.888***	0.142	0.944***	0.810**	−0.005	0.926***	0.426	0.258	0.872***	0.774**	0.395	1	
	Total	0.645*	0.736**	0.942***	0.575	0.859***	0.963***	0.412	0.859***	0.755**	0.652*	0.894**	0.942***	0.799**	0.868***	1

*, ** and*** indicate significance at *p* < 0.05; *p* < 0.01; and *p* < 0.001, respectively.

### 3.6 Correlation among the amount of individual phenolic compounds and TPC, TPhC, TFC, and antioxidant activity

**Figure 3:**
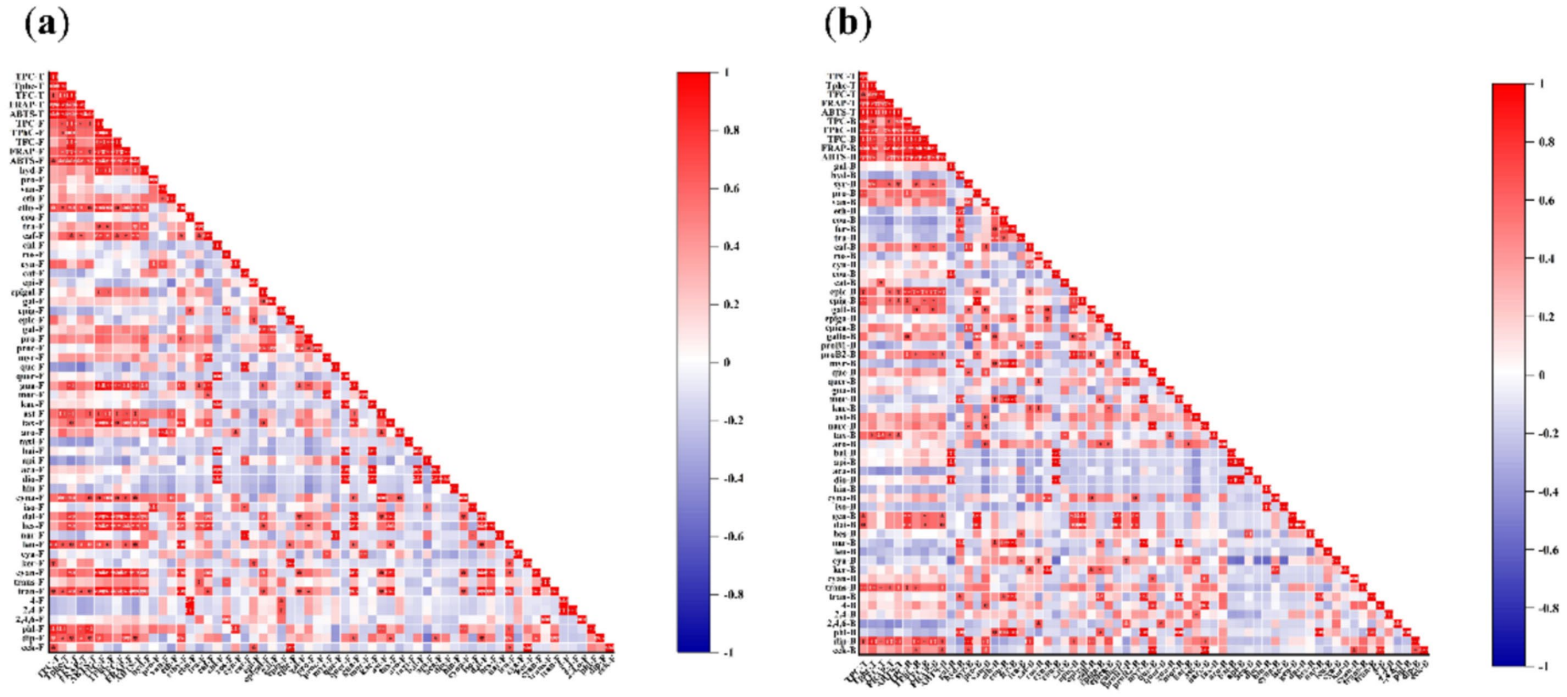
Correlation among the amount of free **(a)** and bound **(b)** individual phenolic compounds and TPC, TPhC, TFC, and antioxidant activity. **(a)** F: free, B: bound, T: total (free plus bound). hyd-F: 2-hydroxybenzoic acid-F, pro-F: protocatechualdehyde-F, van-F: vanillin-F, eth-F: ethyl vanillin-F, ethy-F: ethyl gallate-F, cou-F: p-coumaric acid-F, tra-F: trans-cinnamic acid-F, caf-F: caftaric acid-F, chl-F: chlorogenic acid-F, ros-F: rosmarinic acid-F, cyn-F: cynarin-F, cat-F: catechin-F, epi-F: epicatechin-F, epigal-F: epigallocatechin-F, gal-F: gallocatechin-F, epig-F: epigallocatechin gallate -F, epic-F: epicatechin gallate-F, gal-F: gallocatechin gallate-F, pro-F: procyanidin B1-F, proc-F: procyanidin B2-F, myr-F: myricetin-F, que-F: quercetin-F, quer-F: quercitrin-F, gua-F: guaiaverin-F, mor-F: morin-F, kae-F: kaempferol 3-O-rutinoside-F, ast-F: astragaline-F, tax-F, taxifolin-F, aro-F: aromade0rin-F, taxi-F, taxifolin 7-O-rhamnoside-F, bai-F: baicalein-F, api-F: apigenin-F, aca-F: acacetin-F, dio-F: diosmetin-F, hin-F: hinokiflavone-F, cyna-F: cynaroside-F, iso-F: isovitexin-F, dai-F: daidzein-F, hes-F: hesperidin-F, nar-F: naringenin-F, leu-F: leucocyanidin-F, cya-F: cyanidin-F, ker-F: keracyanin-F, cyan-F: cyanidin-3-O-glucoside-F, trans-F: trans-resveratrol-F, tran-F: trans-piceid-F, 4-F: 4-bromophenol-F, 2,4-F: 2,4-dibromophenol-F, 2,4,6-F: 2,4,6-tribromophenol-F, phl-F: phloroglucinol-F, dip-F: diphlorethol / difucol-F, eck-F: eckol-F. **(b)** F: free, B: bound, T: total (free plus bound). gal-B: gallic acid-B, hyd-B: 2-hydroxybenzoic acid-B, syr-B: syringic acid-B, pro-B: protocatechualdehyde-B, van-B: vanillin-B, eth-B: ethyl vanillin-B, cou-B: p-coumaric acid-B, fer-B: ferulic acid-B, tra-B: trans-cinnamic acid-B, caf-B: caftaric acid-B, ros-B: rosmarinic acid-B, cyn-B: cynarin-B, cou-B: coutaric acid-B, cat-B: catechin-B, epic-B: epicatechin-B, epig-B: epigallocatechin-B, gall-B: gallocatechin-B, epiga-B: epigallocatechin gallate-B, epica-B: epicatechin gallate-B, gallo-B: gallocatechin gallate-B, proB1-B: procyanidin B1-B, proB2-B: procyanidin B2-B; myr-B: myricetin-B, que-B: quercetin-B, quer-B: quercitrin-B, gua-B: guaiaverin-B, mor-B: morin-B, kae-B: kaempferol 3-O-rutinoside-B, ast-B: astragaline-B, narc-B: narcissin-B, tax-B: taxifolin-B, aro-B: aromade0rin-B, bai-B: baicalein-B, api-B: apigenin-B, aca-B: acacetin-B, dio-B: diosmetin-B, hin-B: hinokiflavone-B, cyna-B: cynaroside-B, iso-B: isovitexin-B, gen-B: genistein-B, dai-B: daidzein-B, hes-B: hesperidin-B, nar-B: naringenin-B, leu-B: leucocyanidin-B, cya-B: cyanidin-B, ker-B: keracyanin-B, cyan-B: cyanidin-3-O-glucoside-B, trans-B: trans-resveratrol-B, tran-B: trans-piceid-B, 4-B: 4-bromophenol-B, 2,4-B: 2,4-dibromophenol-B, 2,4,6-B: 2,4,6-tribromophenol-B, phl-B: phloroglucinol-B, dip-B: diphlorethol / difucol-B, eck-B: eckol-B.

Among the free phenolic compounds identified in the seaweeds, the amounts of 2-hydroxybenzoic acid, ethyl gallate, *trans*-cinnamic acid, caftaric acid, epigallocatechin, guaiaverin, astragaline, taxifolin, cynaroside, daidzein, hesperidin, leucocyanidin, cyanidin-3-O-glucoside, trans-piceid, and diphlorethol/difucol were positively correlated with free TPC, TPhC, and TFC (Figure 3a). These phenolic compounds, except for epigallocatechin, were positively correlated with free FRAP and/or ABTS. The amounts of ethyl gallate, caftaric acid, astragaline, cynaroside, leucocyanidin, *trans*-piceid, phloroglucinol, and diphlorethol/difucol positively correlated with total FRAP and ABTS.

Among the bound phenolic compounds identified in the seaweeds, the amounts of syringic acid, protocatechualdehyde, caftaric acid, epicatechin, epigallocatechin, gallocatechin, gallocatechin gallate, procyanidin B_2_, genistein, daidzein, trans-resveratrol, diphlorethol/difucol, and eckol were positively correlated with bound TPC, TPhC, and/or TFC (Figure 3b). These phenolic compounds, except protocatechualdehyde and gallocatechin gallate, showed a positive correlation with bound FRAP and/or ABTS. The amounts of syringic acid, epicatechin, epigallocatechin, taxifolin, *trans*-resveratrol, and diphlorethol/difucol were positively correlated with total FRAP and ABTS.

More importantly, a significant correlation was also shown between the amount of individual phenolic compounds and antioxidant capacity, as indicated by the correlation between free FRAP and free cyanidin-3-O-glucoside (*r* = 0.908, *p* ≤ 0.001), free FRAP and free daidzein (*r* = 0.912, *p* ≤ 0.001), free ABTS and free daidzein (*r* = 0.839, *p* ≤ 0.01), bound FRAP and bound epicatechin gallate (*r* = 0.826, *p* ≤ 0.01), and bound ABTS and bound epicatechin gallate (*r* = 0.822, *p* ≤ 0.01). Therefore, these phenolic compounds may be responsible for the antioxidant activities.

The health properties of phenolics are attributed to their special chemical structure, since they are extremely reactive toward reactive oxygen species due to their electron deficiency (65). Usually, a dihydroxyl group or three adjacent hydroxyl groups on the ring B, and the hydroxyl group in the position of C-5 and C-7 on the ring A are considered as the requirements for antioxidant and antiradical activity of flavonoids (66–68). Many phenolic compounds identified in this study met part or all of these requirements, e.g., cyanidin [(3,4-dihydroxyphenyl)-3,5,7-trihydroxychromenium] and diosmetin (4-methoxy-5,7,3′-trihydroxyflavone). Cyanidin chelated diverse metal ions (such as Fe^2+^, Mg^2+^, and Al^3+^) and thus inhibited the production of reactive oxygen species catalyzed by these metal ions (69). Cyanidin also reduced the catalytic activity of enzymes including the xanthine oxidase enzyme involved in the generation of reactive oxygen species (70). Cyanidin and diosmetin directly scavenged free radicals including superoxide and hydroxyl radicals (29, 71) and modulated Nrf2 antioxidant signaling pathway (70, 72).

### 3.7 Principal component analysis and HCA

**Figure 4:**
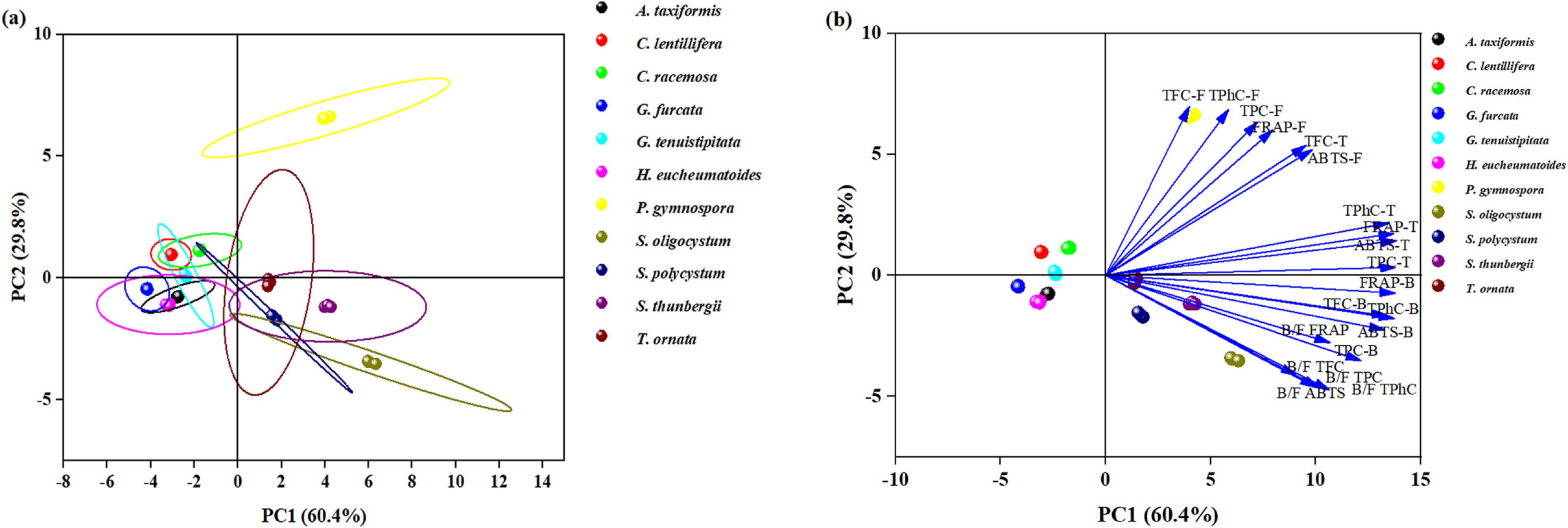
Principal component analysis the free, bound and total TPC, TPhC, TFC, and antioxidant capacity as well as their bound-to-free ratio of different seaweed species. **(a)** Scores plot showing the seaweed clustering, **(b)** Loadings plot reflecting the influence of a particular parameter.

Principal component analysis (PCA) is a multivariate statistical analysis that involves linear transformation of multiple variables, which could reduce the set of factors and identify fewer important variables. This dimensionality reduction approach first reduces the dimensionality of the dataset while maintaining the feature of the dataset with the greatest contribution to variance. Figure 4 and Supplementary Table S1 show the PCA results with the eigenvalue, percentage of variance, and cumulative variance of each variable, and the loadings of PC1 and PC2. PC1 (60.4%) and PC2 (29.8%) with 60.37 and 29.77% of total variance, and provide the Eigen value 12.07, 5.95, respectively. The Eigen value >1 (PC1 and PC2) explaining with 90.14% of total variance.

Seaweeds in the same taxon generally showed similarities in free, bound, and total TPC, TPhC, TFC, antioxidant capacity, and bound-to-free ratio. *P. gymnospora* in the upper right of the PCA had high free TFC, TPhC, TPC, FRAP, ABTS, and total TFC values. In the lower right of the PCA, the other brown seaweeds, except *P. gymnospora*, were distributed with high values of total TPhC, TPC, FRAP, ABTS, bound TFC, TPhC, TPC, FRAP, ABTS, and bound-to-free ratios of TPC, TPhC, TFC, FRAP, and ABTS. All of the green seaweeds (*C. lentillifera* and *C. racemosa*) and red seaweeds (*H. eucheumatoides*, *G. tenuistipitata*, *G. furcata*, and *A. taxiformis*) studied in this paper were distributed in the upper and lower left parts, respectively, with low values of the parameters mentioned above. These results revealed that brown seaweeds may be richer in phenolics, especially bound phenolics, with a more potent antioxidant capacity than green and red seaweeds. García-Casal et al. (32) found that free TPC and TFC in brown seaweeds were higher than those in red and green seaweeds, without the data of bound TPC and TFC, while Sapatinha et al. (30) found a converse result. A great discrepancy in TFC, TPhC, TPC, phenolic profiles, and antioxidant capacity was found among different brown seaweeds, as well as among different red or green seaweeds (1, 31).

**Figure 5:**
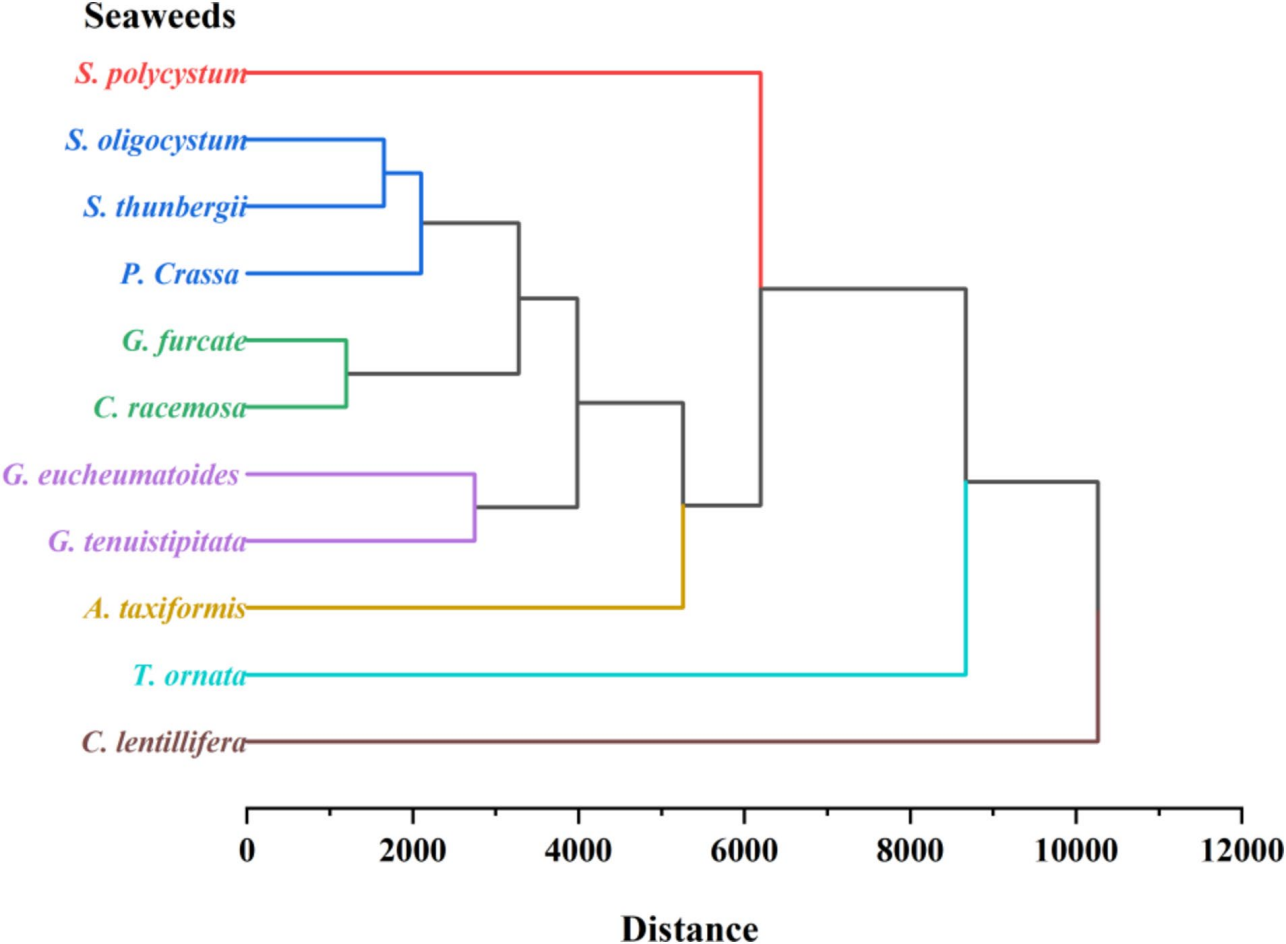
Hierarchical cluster analysis of free, bound, and total TPC, TPhC, TFC, FRAP, ABTS and their bound-to-free ratio, and phenolic profiles of 11 seaweed species.

Among the 11 seaweed species, similarities in free, bound, and total TPC, TPhC, TFC, antioxidant ability, bound-to-free ratio, and phenolic profiles were evaluated using HCA. The seaweeds were divided into seven groups on the HCA map, as indicated by seven lines with different colors (Figure 5). Briefly, brown seaweeds were divided into three groups marked by red, dark blue, and light blue lines. Red and green seaweed were classified into four groups. Phenolics are synthesized via numerous metabolic pathways, and different subclasses of phenolics are usually produced through different pathways (12), which contributes to the difference in the existing form and profile of phenolics (12). The seven groups of seaweeds may have significantly different phenolic metabolic pathways. Previous studies have found that harvesting location and period, genetic factors, and extraction method contribute to the differences in free or bound TPC, TPhC, and TFC of seaweeds (1, 21, 30, 31). The environmental stressors, such as temperature, ultraviolet radiation, nutrient availability, salinity, and desiccation, play an important role in the biosynthesis of phenolic compounds in terrestrial plants (73). In this study, the seaweeds were collected from locations with similar environmental conditions (Hainan Province) and harvested at respective maturity period. Thus, the observed discrepancies in phenolic profiles and consequent antioxidant activity among seaweeds may be mainly attributed to genetic factors. However, three *Sargassum* species (*S. polycystum*, *S. oligocystum*, and *S. thunbergii*) with a highly genetic similarity were divided into two groups on the HCA map. It is generally considered that phenolics play a key role in plants evolution by supplying the specific adaptation and metabolic plasticity to the varying environments (73). The biosynthesis of flavonoids supplied protection against ultraviolet radiation to the terrestrial plants, contributing to the survival under direct sunlight (74). Phlorotannins have been reported to improve the tolerance of plants to osmotic and salinity stress (75), and boost plant growth and overall productivity (76). Throughout the evolutionary course of these seaweeds, the variance of environmental conditions may induce the difference in the biosynthesis of phenolic compounds.

## 4 Conclusion

Significant differences in TPC, TPhC, TFC, antioxidant activity, and phenolic profiles were observed between the free and bound fractions of the 11 seaweed species. *P. gymnospora* had the highest free TPC, free and total TPhC, TFC, and antioxidant activity. *S. thunbergii* exhibited the highest TPhC binding. *S. oligocystum* had the highest bound and total TPC, bound TFC, bound-to-free TPC, TPhC, and TFC ratios. Free TPC, bound TPC, TPhC, and TFC were positively correlated with antioxidant capacity. In total, 17–65 phenolic compounds were found in the seaweed species, with the largest number found in *P. gymnospora*. Coutaric acid and diosmetin were dominant in *S. polycystum*, and hinokiflavone was dominant in *C. lentillifera*, and cyanidin was dominant in the other seaweeds. The HCA divided the 11 seaweed species into seven groups. These results provide useful information for the utilization of seaweed.

## Data Availability

The original contributions presented in the study are included in the article/Supplementary material, further inquiries can be directed to the corresponding author.
