# Supplementary material for: Free and bound phenolic profiles and antioxidant ability of eleven marine macroalgae from the South China Sea
Source: Front Nutr. 2024 Oct 14;11:1459757. doi: 10.3389/fnut.2024.1459757 (PMC11513316; doi:10.3389/fnut.2024.1459757)
Supplement: Supplementary file 1 [file Table_1.DOCX]

Supplementary Material

# Supplementary Data

**Table S1** Loading of parameters for free, bound, and total TPC, TPhC, TFC, FRAP, and ABTS and the bound-to-free ratio of TPC, TPhC, TFC, FRAP, and ABTS on the principal component analysis (PCA) for eleven seaweed species.

| **Variable** | **PC1 Loadings** | **PC2 Loadings** | **Eigen value** | **Percentage of variance (%)** | **Cumulative variance (%)** |
| --- | --- | --- | --- | --- | --- |
| Free TPC | 0.14469 | 0.33755 | 12.07444 | 60.37218 | 60.37218 |
| Bound TPC | 0.2432 | -0.18837 | 5.95361 | 29.76805 | 90.14023 |
| Total TPC | 0.27591 | 0.0172 | 0.78923 | 3.94613 | 94.08636 |
| Free TPhC | 0.11713 | 0.36555 | 0.51692 | 2.58458 | 96.67094 |
| Bound TPhC | 0.26842 | -0.09003 | 0.33595 | 1.67976 | 98.3507 |
| Total TPhC | 0.2693 | 0.11515 | 0.15227 | 0.76136 | 99.11206 |
| Free TFC | 0.07973 | 0.37216 | 0.07409 | 0.37045 | 99.48251 |
| Bound TFC | 0.27479 | -0.09506 | 0.05445 | 0.27224 | 99.75475 |
| Total TFC | 0.19676 | 0.27652 | 0.03034 | 0.15171 | 99.90646 |
| Free FRAP | 0.15927 | 0.32024 | 0.01111 | 0.05556 | 99.96202 |
| Bound FRAP | 0.27556 | -0.03956 | 0.00381 | 0.01906 | 99.98108 |
| Total FRAP | 0.27436 | 0.09211 | 0.00249 | 0.01246 | 99.99354 |
| Free ABTS | 0.19094 | 0.28638 | 8.12536E-4 | 0.00406 | 99.99761 |
| Bound ABTS | 0.26542 | -0.11984 | 3.6944E-4 | 0.00185 | 99.99945 |
| Total ABTS | 0.27703 | 0.07641 | 1.09095E-4 | 5.45476E-4 | 100 |
| B/F TFC | 0.21281 | -0.25271 | 1.16117E-30 | 5.80587E-30 | 100 |
| B/F TPhC | 0.20284 | -0.2471 | 5.12812E-31 | 2.56406E-30 | 100 |
| B/F TFC | 0.18119 | -0.22044 | 2.29837E-31 | 1.14919E-30 | 100 |
| B/F FRAP | 0.21351 | -0.14857 | 1.6229E-31 | 8.11449E-31 | 100 |
| B/F ABTS | 0.19959 | -0.24674 | 1.20804E-31 | 6.04019E-31 | 100 |
